# Multiple Interkingdom Horizontal Gene Transfers in *Pyrenophora* and Closely Related Species and Their Contributions to Phytopathogenic Lifestyles

**DOI:** 10.1371/journal.pone.0060029

**Published:** 2013-03-29

**Authors:** Bao-Fa Sun, Jin-Hua Xiao, Shunmin He, Li Liu, Robert W. Murphy, Da-Wei Huang

**Affiliations:** 1 Key Laboratory of Zoological Systematics and Evolution, Institute of Zoology, Chinese Academy of Sciences, Beijing, China; 2 University of the Chinese Academy of Sciences, Beijing, China; 3 State Key Laboratory of Genetic Resources and Evolution, Kunming Institute of Zoology, Chinese Academy of Sciences, Kunming, China; 4 Centre for Biodiversity and Conservation Biology, Royal Ontario Museum, Toronto, Canada; 5 College of Plant Protection, Shandong Agricultural University, Tai'an, Shandong, China; University of Lausanne, Switzerland

## Abstract

Many studies have reported horizontal gene transfer (HGT) events from eukaryotes, especially fungi. However, only a few investigations summarized multiple interkingdom HGTs involving important phytopathogenic species of *Pyrenophora* and few have investigated the genetic contributions of HGTs to fungi. We investigated HGT events in *P. teres* and *P. tritici-repentis* and discovered that both species harbored 14 HGT genes derived from bacteria and plants, including 12 HGT genes that occurred in both species. One gene coding a leucine-rich repeat protein was present in both species of *Pyrenophora* and it may have been transferred from a host plant. The transfer of genes from a host plant to pathogenic fungi has been reported rarely and we discovered the first evidence for this transfer in phytopathogenic *Pyrenophora*. Two HGTs in *Pyrenophora* underwent subsequent duplications. Some HGT genes had homologs in a few other fungi, indicating relatively ancient transfer events. Functional analyses indicated that half of the HGT genes encoded extracellular proteins and these may have facilitated the infection of plants by *Pyrenophora* via interference with plant defense-response and the degradation of plant cell walls. Some other HGT genes appeared to participate in carbohydrate metabolism. Together, these functions implied that HGTs may have led to highly efficient mechanisms of infection as well as the utilization of host carbohydrates. Evolutionary analyses indicated that HGT genes experienced amelioration, purifying selection, and accelerated evolution. These appeared to constitute adaptations to the background genome of the recipient. The discovery of multiple interkingdom HGTs in *Pyrenophora*, their significance to infection, and their adaptive evolution, provided valuable insights into the evolutionary significance of interkingdom HGTs from multiple donors.

## Introduction

Fungal genus *Pyrenophora* includes almost two hundred species, some of which are important plant pathogens that cause significant losses to crop yields, such as *P. teres*, *P. graminea*, and *P. tritici-repentis*. *Pyrenophora teres*, a necrotrophic fungal pathogen, causes net-spot blotch on economically important agricultural crops such as barley (*Hordeum vulgare*) [1]. *Pyrenophora graminea* is the causal agent of barley stripe, which once caused significant crop yield losses in many areas of the world. Similarly, *P. tritici-repentis* is the agent of tan (or yellow) spot that mainly affects wheat (*Triticum aestivum*); it causes great losses by reducing photosynthesis. The published genomes of two fungal pathogens, *P. teres* and *P. tritici-repentis*, facilitate genetic investigations of their pathogenicity, virulence, and mechanisms of infection, both of which are important factors for combating the negative effects of these fungi.

Horizontal gene transfer (HGT) involves the transfer of genetic information between distantly related organisms outside of normal (vertical) mating systems. HGT is an important driving force in genomic evolution because the recipient acquires novel genes that award new functions. For example, such genes can accelerate adaptation to new environments, broaden host range or change its diet, and even provide the ability to survive in previously lethal conditions [2]. Therefore, HGT represents an important factor in the evolution of species via providing a key source of evolutionary innovations [3].

Well-supported cases of HGT exist, especially in archaeal and eubacterial genomes [4]–[7]. HGT also plays an important role in the innovation and evolution of genomes in fungi and other eukaryotes, although it occurs at a lower frequency compared to prokaryotes [8]. HGT occurs from prokaryotes to fungi and among different species of fungi [9]. In the Fungi Kingdom, recent analyses document the transfer of individual genes [10], gene clusters [11]–[14], and even entire chromosomes [15]–[17]. Such transfers significantly impact the emergence of diseases, niche specification, and/or shifts in metabolic capabilities [18]. HGT can bestow a significant selective advantage to fungi and it may have been more important in fungal evolution than in other eukaryotes [12], [15]. HGT events in some fungi relate to their infection mechanisms because the recipient gains new virulence factors [19]–[20].

One case of HGT in *Pyrenophora* is notable for its recentness; an 11 kb region encoding the host-specific toxin ToxA is thought to have been transferred from *Phaeosphaeria nodorum* (anamorph *Stagonospora nodorum*) to the previously avirulent fungus *P. tritici-repentis* only about 70 years ago [21]–[22]. The gene encoding ToxA also occurs in some isolates of *P. teres*, suggesting a possible, recent HGT between *P. tritici-repentis* and *P. teres* [23]. Apart from this transfer, HGTs are unknown in species of *Pyrenophora*.

Interkingdom HGT, especially bacterial–fungal HGT, plays an important role in the evolution of fungal metabolism, propagation, and pathogenicity [9], [18], [24]. In general, all genes participate in some function and metabolic pathways are well annotated in bacteria and fungi. Most bacterial–fungal HGT genes participate in metabolic pathways and this indicates that they play significant roles in the evolution of fungi [25]. In addition to bacterial–fungal HGT, gene transfers also occur from plants and animals to fungi. These exceedingly rare HGTs add important metabolic traits to the recipient [26]–[27]. To elucidate fully the evolution of *Pyrenophora*, it is necessary to identify potential interkingdom HGT events that may be important to the evolution of phytopathogenic species.

Herein, we explore HGT in a suite of taxa representing a variety of kingdoms ranging from viruses, bacteria, protists, plants, and animals to the fungi *P. teres* and *P. tritici-repentis*. We comprehensively search for homologies and employ phylogenetic analyses. The analyses discover multiple ancient bacterial–fungal HGT events. Some bacterial–fungal HGT genes participate in metabolic pathways and in doing so they add important traits to the recipients. Other HGTs involving protein-coding genes associated with extracellular locations may advance virulence. A probable HGT gene coding a leucine-rich repeat protein in two species of *Pyrenophora* may originate from a host plant; this gene may play an important role in plant pathogenesis and host protein–protein interactions. Homology distributions of the HGT genes derived from bacteria and plants, taken together with their compositional characteristics and conservative synteny in fungal recipients, identify some ancient HGT events. Evolutionary analyses identify amelioration, purifying selection, and accelerated molecular evolution in these HGT genes, indicating adaption to new genomic backgrounds. Ours is the first thorough survey of HGT in species of *Pyrenophora* and their closely related species using analyses of whole genomes. It opens the first window to understanding the role interkingdom HGT plays in the evolution of these fungi.

## Results and Discussion

### Both *P. teres* and *P. tritici-repentis* Possess 14 HGT Genes

**Figure 1:**
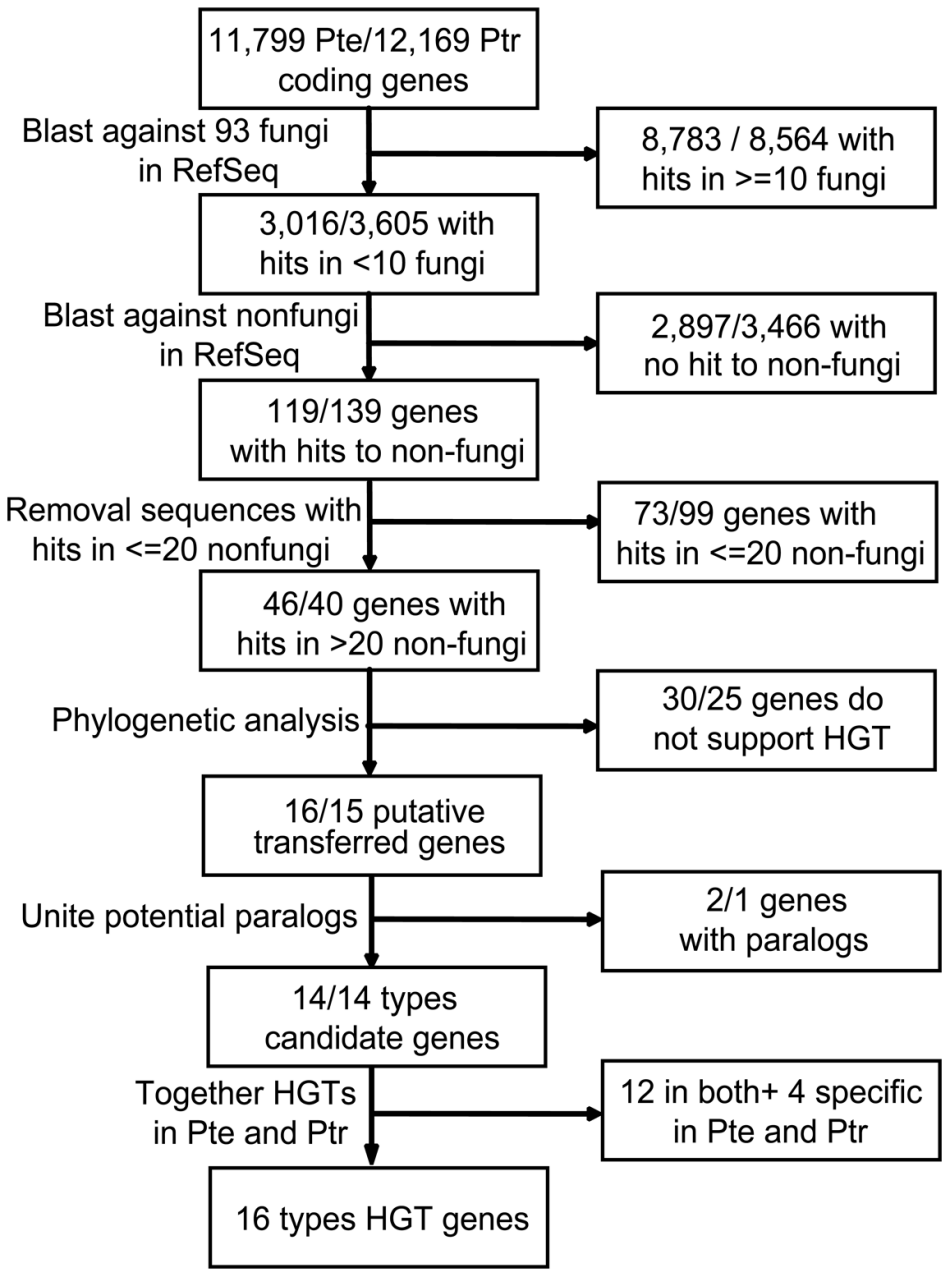
Flow chart of the screening methods used to detect the horizontally transferred genes and the results of each step. Pte represents *Pyrenophora teres* and Ptr represents *Pyrenophora tritici-repentis*.

We used a series of stringent filters to identify promising HGT events in *P. teres* and *P. tritici-repentis* (Figure 1). The first filter identified protein-homologs occurring in fewer than 10 of 93 fungal taxa. It identified 3016 and 3605 candidate protein-coding genes in *P. teres* and *P. tritici-repentis*, respectively. The second filter, which required a high level of similarity to more than 20 non-fungal organisms, was applied to these candidate genes. It identified 46 and 40 genes in *P. teres* and *P. tritici-repentis*, respectively, as having a high possibility of being acquired via HGT. The third filter, which used phylogenetic analyses, validated 16 genes as being reliable candidate HGTs in *P. teres* and *P. tritici-repentis* together. Among these genes, both species had 14 HGT genes, 12 of which occurred in both species, meaning that each of them owned 2 specific HGT genes (Table 1). ML and BI phylogenies and NJ trees validated the HGTs; all three methods obtained similar trees (Figure S1). Unfortunately, limited EST data for both species precluded analyses of expression.

**Table 1:** The 16 candidate HGT genes in the genomes of *P. teres* and *P. tritici-repentis*.

	Gene ID (*P. teres*)	Gene ID (*P. tritici-repentis*)	Other fungi with HGT genes	Gene name	Top hit species	E-value	Similarity
1	XP_003306939.1	XP_001937118.1		leucine-rich repeat protein	*Hordeum vulgare*	3e-60	47%
2		XP_001942153.1		methyltransferase MppJ	*Streptomyces hygroscopicus*	5e-141	59%
3		XP_001937843.1	*Phaeosphaeria nodorum Zymoseptoria tritici*	beta-galactosidase	*Bryantella formatexigens*	0	39%
4	XP_003299971.1	XP_001934409.1	*Phaeosphaeria nodorum Leptosphaeria maculans*	UDP-glucosyltransferase	*Clostridium beijerinckii*	8e-98	39%
5	XP_003296325.1	XP_001932301.1	*Phaeosphaeria nodorum Leptosphaeria maculans*	GCN5-related N-acetyltransferase	*Candidatus Koribacter versatilis*	4e-48	47%
6	XP_003304278.1	XP_001940400.1	*Phaeosphaeria nodorum Leptosphaeria maculans*	oxidoreductase, Gfo/Idh/MocA family	*Listeria innocua*	7e-97	48%
7	XP_003305055.1 XP_003297111.1	XP_001930965.1	***Phaeosphaeria nodorum Leptosphaeria maculans***	enterochelin esterase-like enzyme	*Mesotoga prima*	3e-85	37%
8	XP_003300109.1	XP_001938894.1	*Fusarium oxysporum Nectria haematococca*	N-acetylglucosaminyltransferase	*Desulfobacca acetoxidans*	2e-55	38%
9	XP_003305149.1	XP_001940578.1	*Phaeosphaeria nodorum Leptosphaeria maculans Zymoseptoria tritici*	succinylglutamate desuccinylase/aspartoacylase	*Laribacter hongkongensis*	3e-69	36%
10	XP_003299132.1	XP_001937221.1	*Phaeosphaeria nodorum Leptosphaeria maculans Botryotinia fuckeliana*	5-formyltetrahydrofolate cyclo-ligase	*Rhizobium* sp.	5e-55	43%
11	XP_003299846.1	XP_001935333.1	*Phaeosphaeria nodorum Verticillium albo-atrum Verticillium dahliae*	NmrA family protein	*Maritimibacter alkaliphilus*	1e-71	42%
12	XP_003296129.1 XP_003306041.1	XP_001935567.1 XP_001933875.1	*Phaeosphaeria nodorum Leptosphaeria maculans Zymoseptoria tritici Exophiala dermatitidis*	glcG protein	*Roseovarius* sp.	5e-35	53%
13	XP_003299438.1	XP_001935182.1	*Phaeosphaeria nodorum Leptosphaeria maculans Gaeumannomyces graminis Chaetomium globosum Gibberella zeae*	xylanase A	*Streptomyces scabiei*	4e-144	41%
14	XP_003304690.1	XP_001938001.1	*Phaeosphaeria nodorum Zymoseptoria tritici Trichoderma atroviride Trichoderma reesei Trichoderma virens*	cyanophycinase	*Spirosoma linguale*	1e-76	47%
15	XP_003297280.1		*Phaeosphaeria nodorum Magnaporthe oryzae Penicillium marneffei Talaromyces stipitatus*	alpha/beta hydrolase	*Bradyrhizobium* sp.	1e-39	34%
16	XP_003305619.1		*Phaeosphaeria nodorum Verticillium albo-atrum Verticillium dahliae Colletotrichum higginsianum Glomerella graminicola Gaeumannomyces graminis*	oxidoreductase	*Kribbella flavida*	2e-102	51%

*Pyrenophora teres* represents two species in the genus and is used to get top-hits species, e-values, and similarity values. Other fungal species were removed from top-hit organism.

**Figure 2:**
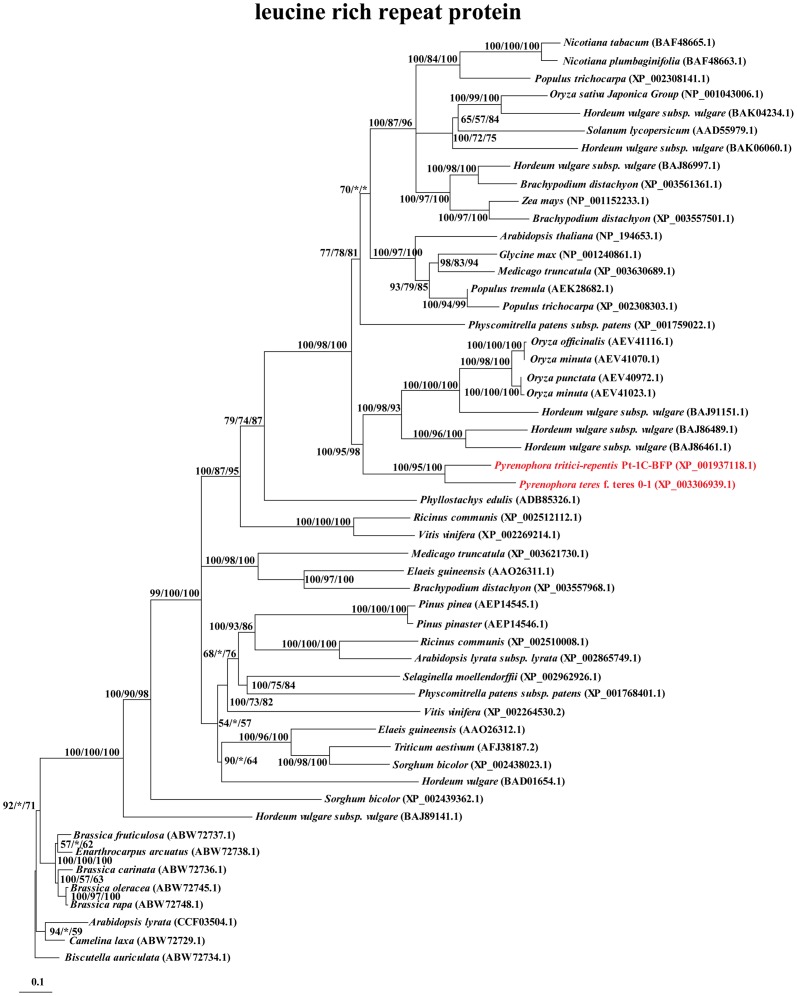
Phylogeny of the HGT gene leucine-rich repeat protein in *Pyrenophora*. The Bayesian inference tree (shown) is virtually identical to maximum likelihood and neighbor-joining trees. Nodal support values ≥50 shown (BI/ML/NJ). Asterisks (*) indicate support values <50. Scale bar indicates substitutions per site. Sequences from *Pyrenophora* are indicated in red.

Ours is the first study to report interkingdom HGT events for two species of *Pyrenophora*. The 16 HGT genes are novel and unreported previously. Of particular interest, a gene encoding leucine-rich repeat protein appears to be a transfer from the host barley, *Hordeum vulgare* (Figure 2). The host plant may have multiple copies of this gene, one of which now occurs in *Pyrenophora*. The transfer of the leucine-rich repeat protein may promote the infection of plant hosts by *Pyrenophora*. HGT from host plants to fungi is rarely reported and our result provides a good example of it [26].

Bacteria donate most of the HGT genes; the leucine-rich repeat protein is the only gene from a plant. Some bacterial donors, such as *Rhizobium* sp. and *Bradyrhizobium* sp., also associate with plants. Sympatry may facilitate the exchange of genetic material between them and *Pyrenophora* [28]. No evidence suggests HGT between *Pyrenophora* and any species of animal, virus, or protist.

Several lines of evidence eliminate the possibility of contamination from bacteria and plants, which could explain our results. First, all other HGT genes occur in some other fungal species in addition to *Pyrenophora*, except for methyltransferase MppJ and leucine-rich repeat protein, which are only present in one and two species of *Pyrenophora*, respectively. The widespread occurrence of most HGT genes precludes contamination from bacteria or plants [29]. Second, all flanking sequences of these HGT genes are quite similar to and appear to be fungal sequences. These data are consistent with vertical inheritance where HGT genes occur in the genome of *Pyrenophora* and physically link to native fungal sequences [29]. Third, many of these genes have few or no introns (Table S1) and this pattern is consistent with HGT from bacteria. The percentages of HGT genes with introns in the complete genomes of *P. teres* and *P. tritici-repentis* are 73.08% and 78.17% respectively. Intron density, i.e. the number of introns per HGT gene in the complete genome, is 1.49 in *P. teres* and 1.69 in *P. tritici-repentis*. This background distribution, which indicates a large average number of introns in the genomes of *Pyrenophora*, further supports the likelihood of HGT. Taken together these three lines of evidence confirm the authenticity of the HGT genes.

Among the 16 HGT genes, two have divergent paralogs. Enterochelin esterase-like enzyme has two copies in *P. teres*, but only one copy in *P. tritici-repentis* (Figure S2). Further, GlcG protein has two copies in both species of *Pyrenophora* as well as in the fungi *Phaeosphaeria nodorum* and *Leptosphaeria maculans*. The genes trees indicate that the acquisition of the second copies occurred via post-transfer gene duplications. No pseudogenes occur for any of the 16 HGT genes in the two genomes of *Pyrenophora* and this may relate to their functional importance.

### Evolutionary Mapping of HGT Events

Gene losses may explain all 16 candidate HGT events. This scenario requires an ancient origin of the genes during the early diversification of eukaryotes followed by multiple losses. This is highly unlikely for several reasons. First, the narrow taxonomic distribution of the putative HGT genes makes gene silencing unlikely because of the numerous independent gene loss events required for all other lineages sampled [26]. Second, this scenario cannot explain the high level of sequence identity between species of *Pyrenophora* and their candidate donors (Table 1) [12]. Third, our phylogenetic analyses nest each HGT gene within the donor group with strong nodal support (Figure S1). A massive, independent loss of genes is extremely complicated and less parsimonious (less likely) than the acquisition of novel genes via HGT [9]. The HGT scenario is much more consistent and parsimonious with the observed patterns of the 16 HGT genes [26].

The taxonomic distribution of HGT genes in fungi indicates both ancient and recent HGT events in *Pyrenophora*. We assume the most parsimonious scenario when mapping HGT events on the fungal tree of life [24]. Both species of *Pyrenophora* have two HGT genes not present in the other. The gene encoding methyltransferase MppJ occurs only in *P. tritici-repentis*, indicating a recent gene transfer event after divergence from *P. teres*. The occurrence of beta-galactosidase *P. tritici-repentis* and other fungi but its absence in *P. teres* indicates an ancient transfer and subsequent loss in *P. teres*. Genes encoding alpha/beta hydrolase and oxidoreductase occur in *P. teres* but not *P. tritici-repenti*. Their presence in other fungi indicates transfer of these genes in a common ancestor and subsequent losses in *P. tritici-repentis*. Transferred from a plant, leucine-rich repeat protein occurs only in *Pyrenophora*; this distribution implies that its transfer event occurred sometime after the formation of *Pyrenophora*.

**Figure 3:**
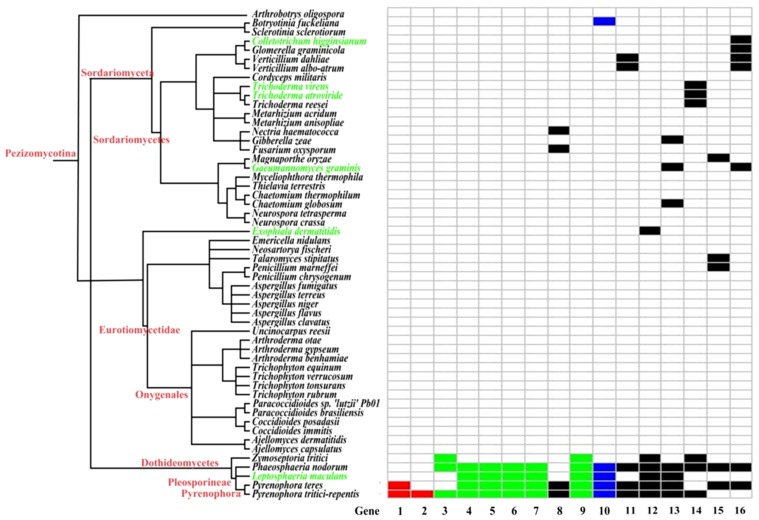
The presence or absence of HGT genes in fungi. Species tree constructed from the taxonomy database of NCBI along with superfamily membership. Species with HGT genes but without genomic data are indicated in green. Boxes indicate possession of the gene. Red boxes: Genes limited to *Pyrenophora*, indicating a recent gene transfer event after divergence of *Pyrenophora*. Green boxes: Genes limited to Dothideomycetes, indicating a relatively ancient gene transfer event after divergence of Dothideomycetes. Blue boxes: Genes form two fungus clades that nest within non-fungal species in the corresponding gene tree, indicating two independent interkingdom transfers. Black boxes: Genes were present in *Pyrenophora* and distantly related organisms. Gene trees indicated independent interkingdom transfers. The trees cannot determine the presence of fungi–fungi HGT. Genes 1–16 refer to the genes encoding leucine-rich repeat protein, methyltransferase MppJ, beta-galactosidase, UDP-glucosyltransferase, GCN5-related N-acetyltransferase, oxidoreductase, Gfo/Idh/MocA family, enterochelin esterase-like enzyme, N-acetylglucosaminyltransferase, succinylglutamate desuccinylase/aspartoacylase, 5-formyltetrahydrofolate cyclo-ligase, NmrA family protein, glcG protein, xylanase A, cyanophycinase, alpha/beta hydrolase, oxidoreductase, respectively.

Most of the HGT genes are present in species of *Pyrenophora*, *L. maculans*, and *Phaeosphaeria nodorum*, all of which belong to the Pleosporineae. This distribution indicates that HGT occurred before diversification of these fungi (Figure 3). Previous studies indicated that mesosynteny is rampant in Pleosporineae and could mask gene order evidence. Nevertheless, the detected HGT genes generally exhibit conserved synteny between them (Table S2). This pattern indicates a single HGT event and not multiple independent transfers to them [30].

Some HGT genes occur not only in lineages of *Pyrenophora* and in other species in the Dothideomycetes but also in distantly related organisms. Except for 5-formyltetrahydrofolate cyclo-ligase, genes the fungi form clades that then nest within non-fungal species in the respective gene trees. Each incident represents an independent interkingdom transfer in a common ancestor, which is followed by subsequent losses in some taxa. The possibility of fungi–fungi HGT cannot be tested because of too few fungal species other than Dothideomycetes have these HGTs. In general, congruence between the relative positions of fungal lineages and the taxonomy supports the absence of fungi–fungi transfers [31]. However, the number of fungal species in the trees precludes testing for this.

Thirteen of the 16 HGTs in the Pezizomycotina (excluding glcG protein, cyanophycinase, and alpha/beta hydrolase) associate with plants. Except for *Sclerotinia sclerotiorum* 1980 UF-70, all other fungal species associating to plants in Pezizomycotina with genome data share certain HGT genes with phytopathogenic *Pyrenophora* (Table S3). Thus, HGT genes seem to facilitate or enhance phytopathogenicity. Some recipients, such as *Gaeumannomyces graminis*, *Magnaporthe oryzae*, and *Gibberella zeae*, infect crops in the Gramineae. The HGTs in *Pyrenophora* may be functional equivalents of those in other fungi by facilitating the ability to infect similar hosts. The transfer of genes to phytopathogenic species is known; two novel, virulent bacteria-derived HGT genes occur in fungal pathogens of cereals, but are absent in non-cereal pathogens [28].

### Extracellular HGT Coding-proteins

COG, GO, and KEGG analyses were used to predict the function of each HGT gene (Table 2). Unfortunately, the COG and KEGG databases have only six and two HGT genes, respectively, for which functional and metabolic network data are available. Regardless, COG categories indicate primary involvement in the transport and metabolism of carbohydrates and coenzymes. Some HGT genes functionally relate to biogenesis of the cell wall/membrane/envelope. GO analysis indicate that HGT genes have diverse functions. Some molecular functions involve hydrolase, transferase, oxidoreductase, and catalytic activities. Further, xylanase A and cyanophycinase take part in the catabolic process and proteolysis of xylan, respectively. Some HGT gene products bind proteins, carbohydrates, esters, ATPs, and nucleotides. In the KEGG database, beta-galactosidase participates in the metabolism of starch and sucrose, and glycosaminoglycan degradation; 5-formyltetrahydrofolate cyclo-ligase relates to one folate carbon pool (Table 2).

**Table 2:** COG, GO, and KEGG biochemical pathway mappings and the predicted cellular locations of HGT genes.

	Gene name	COG categories	GO categories	KEGG pathways	TargetP	SignalP	TMHMM	WoLF PSORT
1	Leucine-rich repeat protein	Unknown	protein binding	Unknown	S	YES	-	**extr**
2	methyltransferase MppJ	Unknown	methyltransferase activity	Unknown	-		-	cyto
3	beta-galactosidase	Carbohydrate transport and metabolism	hydrolase activity, catalytic activity	Starch and sucrose metabolism Glycosaminoglycan degradation	-		-	mito
4	UDP-glucosyltransferase	Carbohydrate transport and metabolism Energy production and conversion	transferase activity, carbohydrate binding, lipid glycosylation	Unknown	-		-	cyto
5	GCN5-related N-acetyltransferase	Unknown	N-acetyltransferase activity	Unknown	-		-	cyto
6	oxidoreductase, Gfo/Idh/MocA family	Carbohydrate transport and metabolism Secondary metabolites biosynthesis Transport and catabolism	oxidoreductase activity, oxidation-reduction process	Unknown	-		-	cyto
7	enterochelin esterase-like enzyme	Unknown	Unknown	Unknown	S	YES	-	**extr**
8	N-acetylglucosaminyltransferase	Cell wall/membrane/envelope biogenesis	transferase activity	Unknown	S	YES	-	**extr**
9	succinylglutamate desuccinylase/aspartoacylase	Unknown	hydrolase activity, ester bonds	Unknown	S	YES	-	**extr**
10	5-formyltetrahydrofolate cyclo-ligase	Coenzyme transport and metabolism	folic acid-containing compound biosynthetic process, formyltetrahydrofolate cyclo-ligase activity, ATP binding	One carbon pool by folate Metabolic pathways	-		-	cyto
11	NmrA family protein	Carbohydrate transport and metabolism Cell wall/membrane/envelope biogenesis	nucleotide binding	Unknown	M		-	mito
12	glcG protein	Unknown	Unknown	Unknown	-		-	mito
13	xylanase A	Unknown	xylan catabolic process	Unknown	S	YES	-	**extr**
14	cyanophycinase	Unknown	serine-type peptidase activity, proteolysis	Unknown	S	YES	-	**extr**
15	alpha/beta hydrolase	Unknown	catalytic activity	Unknown	M		-	mito
16	oxidoreductase	Unknown	Unknown	Unknown	S	YES	-	**extr**

Genes without definite information in the database were indicated as being unknown. Genes participating in multiple functions and biochemical pathways were shown. In the sixth column, S = secretory pathway, M = to mitochondrion location, and – = any other location. In the seventh column, YES = sequence has signal peptide. In the eighth column, – = HMM not present. In the last column, extr, cyto, and mito refer to protein likely localized in extracellular sites (bold font), cytoplasm, and mitochondria, respectively.

It is surprising that seven, about half of the 16 HGT genes have putative extracellular locations. Extracellular enzymes may relate to interactions between species of *Pyrenophora* and their host plants. This is especially true for leucine-rich repeat protein, whose gene may be from the host plant itself.

Genes with nucleotide binding sites (NBS), leucine-rich repeats (LRR) and/or serine/threonine protein kinase (S/TPK) domains often confer plant disease-resistance. Tsn1 has disease-resistance gene-like features, including S/TPK and NBS-LRR domains in host plants [32]. The gain and secretion of host LRR protein by species of *Pyrenophora* may interfere with Tsn1–ToxA interactions. Unfortunately, the absence of EST data for leucine-rich repeat protein precludes analyses of variation in expression during the process of infection. Although we cannot demonstrate its function directly, the LRR domain, protein-binding function, and extracellular location indicate that leucine-rich repeat protein may facilitate interaction of the two species of *Pyrenophora* and their hosts. In some plants, this protein appears to function as receptor-like kinase or disease resistance protein. Faris et al. suggested that species of *Pyrenophora* may thrive by subverting the resistance mechanisms acquired by plants to combat other pathogens [32]. We suspect that leucine-rich repeat protein subverts host resistance [32].

Among the other six HGT genes with extracellular locations, xylanase A functions in xylan catabolism. This function is consistent with the necrotrophic lifestyle of phytopathogenic species of *Pyrenophora*. Xylanase is an important constituent of the plant's cell wall, and xylanase A can interact with it to promote the degradation of the structure. Species of *Pyrenophora* and other fungi acquired the gene encoding xylanase A from bacteria and they secrete the enzyme extracellularly. The phyletic distributions of the recipients indicates that all fungi with this gene associate with plants (Table 1).

Cyanophycinase is involved in the processing of proteolysis. N-acetylglucosaminyl transferase and succinylglutamate desuccinylase/aspartoacylase perform transferase and hydrolase activities, respectively; the former also participates in cell wall/membrane/envelope biogenesis and the latter can bind esters. We suspect the infection of plants by *Pyrenophora* also involves these enzymes, although adequate functional information is wanting. Little functional information also exists for enterochelin esterase-like enzyme and oxidoreductase. Nevertheless, the duplication of the former enzyme in *P. teres* indicates that its encoding HGT gene may play an important role.

Some genes that encode intracellular proteins appear to have common, specific functions related to phytopathogenicity. For example, beta-galactosidase and UDP-glucosyltransferase are involved in metabolic steps associated with pectin degradation. However, no phylogenetic and genomic linkage evidence suggests that these two genes were acquired simultaneously to confer a larger function. No other candidate HGTs show coupled functions or are involved in the same pathway.

Alpha/beta hydrolase regulates the interactions between pathogenic bacteria and their hosts, indicating its potential importance in the phytopathogenicity of species of *Pyrenophora* [33]. Some members of GCN5-related N-acetyltransferase confer resistance to aminoglycoside antibiotics in certain bacteria [34]. The presence of this gene may increase the resistance of *Pyrenophora* to aminoglycoside antibiotics.

Both phytopathogenic species of *Pyrenophora* have HGT genes that relate to functional requirements of the recipients. These not only promote infection and pathogenicity, but also participate in the metabolism of carbohydrates.

### Compositional Similarity of HGT and Recipients Genes

At the time of transfer, HGT genes reflect the base composition of the donor genome. Over time these sequences will conform to the nucleotide composition of the new genome because HGT genes are subject to the same mutational processes affecting all genes in the recipient genome. This process is known as amelioration [35].

The extent of amelioration, if any, of HGT genes was determined by combining analyses involving G+C content with patterns of codon usage. Similarities of GC content and codon usage bias among HGT genes and recipients can indicate their amelioration and suggest adaptation of HGT genes to recipient genomes [24]. In *P. tritici-repentis*, GC3s and overall GC content of most HGT genes display a more centralized distribution and higher similarity to the recipient than species in the donor group (Figure S3). Analyses of codon usage bias also indicate amelioration. All four indices of HGT genes—CAI, CBI, FOP, and ENC—resolve greater similarity to the background genome of the recipient (Figure S4). Results of the compositional analyses of HGT genes in *P. teres* and *P. tritici-repentis* parallel each other. These patterns indicate that transferred genes are adjusting in composition to match the new genomes. Amelioration may relate to the roles played by HGT genes in the genomic background of the recipient. They must adapt to this new background and in doing so they modify the original sequence composition to obtain a new composition similar to that of the recipient. This pattern also helps to eliminate the possibility erroneously identifying HGT genes owing to contamination.

The extent of amelioration is a function of antiquity of the HGT event. Most of the HGT events were ancient and amelioration appears to be complete or nearly so. In contrast, leucine-rich repeat protein has values such as for CBI and Fop that are more similar to the host plant than to the background of the fungi. In this case, the HGT genes do not fit the new genome well and this discovery suggests a recent transfer event.

### Purifying Selection and Accelerated Evolution of HGT Genes

**Figure 4:**
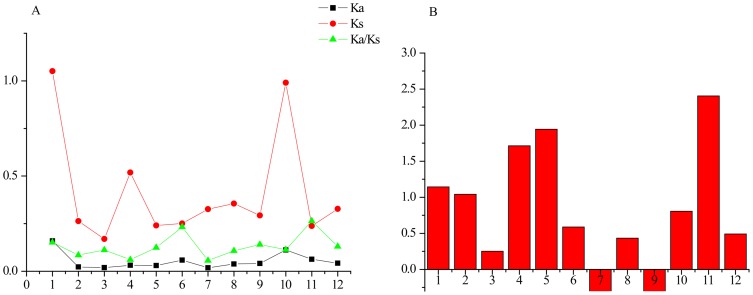
Selection pressure and evolutionary rates of HGTs in species of *Pyrenophora*. (A) Nonsynonymous (Ka) and synonymous (Ks) mutations and their ratio Ka/Ks for the HGTs among species of *Pyrenophora*. (B) Comparison of molecular evolutionary rates between transferred and corresponding donor genes. Ka/Ks values of HGT genes in species of *Pyrenophora* were divided by Ka/Ks values of their corresponding homologs in donor group species, and the distribution of the log ratios was plotted. Values less than 0 indicated that transferred genes had lower Ka/Ks values than the corresponding donor genes. Values greater than 0 indicated that transferred genes had higher Ka/Ks values than corresponding donor genes. Genes 1–12 refer to genes encoding leucine-rich repeat protein, UDP-glucosyltransferase, GCN5-related N-acetyltransferase, oxidoreductase, Gfo/Idh/MocA family, enterochelin esterase-like enzyme, N-acetylglucosaminyltransferase, succinylglutamate desuccinylase/aspartoacylase, 5-formyltetrahydrofolate cyclo-ligase, NmrA family protein, glcG protein, xylanase A, cyanophycinase, respectively.

In analyses of selection pressure, Ka/Ks values of less than 1 indicate negative selection on gene sequences and values of greater than 1 indicate positive selection. Ka/Ks values for most HGT genes are less than 0.5, indicating negative selection; no signals of positive selection occur for the HGT genes of *Pyrenophora*. For example, Figure 4A shows values of Ka, Ks, and Ka/Ks for the 12 HGT genes in both recipient species of *Pyrenophora*. The synonymous mutation may relate to fine tuning of nucleotide composition to fit a new genomic background. Most variation occurs in the third codon position. Variation in this position rarely influences function. Evolution of this position primarily changes overall nucleotide composition. Because Ka/Ks values may reflect protein functions, low values suggest that HGT genes experienced purifying selection. This discovery also indicates fully functional HGT genes [36].

Branch- and site-models also do not detect signals of positive selection. Positive selection can occur in multigenic families produced by duplication events [37]. However, both HGT genes with paralogs do not have signals of positive selection.

A comparison of Ka/Ks values for each HGT gene in both recipient species of *Pyrenophora* and in their corresponding donor groups can identify accelerated molecular evolution following HGT. Our analysis finds significantly higher Ka/Ks values of HGT genes in fungal recipients compared to donor groups (*P*<0.001) (Figure 4B). This discovery indicates accelerated evolution of recipient HGT genes. Similar results occur for HGT genes in *P. teres*. Accelerated molecular evolution may occur in HGT genes upon their disruption and loss of function [38]. However, our results indicate that functional HGT genes also undergo accelerated molecular evolution, likely for adaption to their new genomic backgrounds.

## Conclusions

Multiple interkingdom HGT occurs in species of *Pyrenophora* and donors include a diversity of bacteria and host plants. This is the first report of probable HGT from host plants to species of *Pyrenophora*. Seven HGT genes encode extracellular proteins that may drive the infection of host plants by species of *Pyrenophora* and closely related species. Some of these proteins likely interfere with plant defense-response. Other enzymes participate in the degradation of plant cell walls. Further, some intracellular enzymes encoded by HGT genes may participate in carbohydrate metabolism. Therefore, the HGT genes undoubtedly contribute to virulence and the utilization of their carbohydrates of host plants by species of *Pyrenophora*. Amelioration, purifying selection, and accelerated molecular evolution adapted HGT genes to new genomic backgrounds. Multiple interkingdom horizontal gene transfers may make significant contributions to the evolution of phytopathogenic *Pyrenophora* and their closely related species. Further investigations will lead to a more complete understanding of the biological functions of these HGT genes.

## Materials and Methods

### Sequence Sources

The genomic sequences of *P. teres* and *P. tritici-repentis*, which have 33.58 and 37.36 million base pairs (Mbp) with 11,799 and 12,169 identified protein-coding genes, respectively [1], were downloaded from NCBI. All coding-genes and predicted protein sequences were also downloaded from NCBI.

The RefSeq and non-redundant (nr) sequences (2012.6.21) were downloaded from NCBI (http://www.ncbi.nlm.nih.gov/) along with the corresponding taxonomy (http://www.ncbi.nlm.nih.gov/taxonomy). This data contained a wide diversity of viral, eukaryotic, and prokaryotic taxa in the following groups: viruses, bacteria, fungi, protozoans, plants, and animals.

Two local proteome databases were constructed for analysis. One was a comprehensive fungal proteome database containing all proteins of 93 fungal taxa with completed genomes in RefSeq (Table S3). The other database contained all proteomes downloaded from RefSeq excluding fungal sequences.

### Search Pipeline for HGT

Clues as to the occurrence of HGTs and their directionality were inferred from species-distributions on gene trees. HGT genes were assumed to have a narrow taxonomic range within its group (e.g., fungi) but a wide taxonomic diversity in other groups (e.g., plants, bacteria) [39].

To identify possible HGT events in *P. teres* and *P. tritici-repentis*, we used a multistep, bioinformatic pipeline. Each genome of *Pyrenophora* was treated separately. We used BLAST to detect proteins present in a few fungal species only but widespread in other kingdoms [40] based on the protocols of Marcet-Houben and Gabaldon for their research on HGTs from prokaryotes to fungi [24]. First, each predicted protein query-sequence was compared against fungal sequences in the local database using BLASTP with an e-value threshold of 1e^−5^. We aimed to detect recent HGT events involving species of *Pyrenophora* only as well as to identify ancient HGTs in *Pyrenophora* and other fungi. Therefore, proteins found in less than 10 fungal species were considered to be candidates. Next, candidate protein-sequences were compared to the non-fungi database using BLASTP with an e-value threshold of 1e^−5^. Proteins with over 20 species-hits in non-fungi were selected for further study [24]. Except for a few fungal hits, these sequences had high similarity to non-fungal species and were indicated to be transferred from them. We constructed gene trees (see below) for each retained protein and its homologs to validate the reliability of HGT. The detailed pipeline was shown in Figure 1.

The amino acid (AA) lengths and number of introns of candidate HGT genes in the genomes of *P. teres* and *P. tritici-repentis* we were calculated. We searched the transferred proteins in *Pyrenophora* against their own corresponding genome using a TBLASTN (e-value cutoff 10^−10^) to identify potential pseudogenes, those that could not translate intact to protein sequences.

We tested for possible artifacts and contamination in the two species of *Pyrenophora*. HGT genes present in a single genome of *Pyrenophora* were tested for being a product of DNA contamination from other organisms during genome-sequencing. When flanking sequences of HGT gene indicated vertical inheritance by being congruent with the species phylogeny, we assumed the putative HGT gene was located on the genome of *Pyrenophora* and physically linked to native genes [29].

### Phylogenetic Analyses

We reconstructed gene trees for all retained candidate HGT genes including their closest homologs. Homologs were identified by searching non-redundant databases by BLASTP with an e-value 1e^−5^. The selected sets of significant homologous proteins were subjected to multiple alignment with ClustalW2 [41]. The alignments were inspected visually and refined manually.

Phylogenetic analyses were performed with two approaches: maximum likelihood (ML) and Bayesian inference (BI). We also employed distance-based neighbor-joining (NJ) trees, which were constructed by using the program Neighbor in Mega5 [42]. Bootstrap support values were obtained by generating 1,000 pseudo-replicates. ML phylogenies were constructed by Phyml [43] using the best-fit evolutionary model as suggested by Prottest 3.0 [44]. In all cases, a discrete gamma-distribution model with four rate-categories plus invariant positions was used. The gamma parameter and proportion of invariant sites were estimated from the data. Branch support values were obtained by 1,000 bootstrap pseudoreplicates. BI phylogenies were generated by MrBayes 3.1.2 [45]. For each HGT gene, we ran two independent analyses using four Metropolis-coupled Monte Carlo Markov Chains (MCMC), each with one cold and three heated chains for one million generations while sampling every 100 generations. We stopped the runs when the average deviation of split frequencies reached less than 0.01. We sampled trees every 100 generations and discarded the initial 25% of the total trees as burn-in. Compatible groups were shown in the majority rule consensus tree; the frequency of nodal resolution was assumed to indicate reliability. HGT was identified when few fungal sequences nested within many non-related species on a well-supported node.

### Evolutionary Mapping of HGT Events

When a transferred gene occurred in a single species of *Pyrenophora*, the transfer was assumed to have occurred in that specific lineage only. Candidate genes that had hits in two species of *Pyrenophora* indicated a HGT event in their common ancestor. When candidate HGT genes had homologs in several fungal species that formed a monophyletic group and then nested in non-fungal species, we assumed that the common ancestor of all fungal species received the HGT. Multiple HGT events from a similar donor with subsequent transfer among fungi was also possible. In this case, we analyzed the tree topology and investigated synteny of each HGT gene across both species of *Pyrenophora* and other fungi. If the homologs did not form a monophyletic group, the protein could have been transferred via multiple independent HGTs [24].

### Functional Assignments of HGT Genes

We performed COG, GO, and KEGG analyses to infer function. KEGG analyses inferred that HGT proteins functioned within the metabolic network. For this analysis, we used KAAS (KEGG automated annotation server) to query metabolic pathways of HGT genes by BDH (bidirectional hit) orthology searches [46]. In addition to gene annotation searches, we also surveyed the literature.

To investigate putative cellular locations of each HGT-encoded protein, all HGT genes in both species of *Pyrenophora* were subjected to SignalP [47], TargetP [48], TMHMM [49] and WoLFPSORT [50] analyses with default settings. These methods predicted which genes among all possible candidates secreted proteins along with possible secretion signals and/or transmembrane domains. Secretion was predicted for proteins that 1) had a signal peptide (SignalP), 2) were predicted to be secreted (TargetP), and 3) had no transmembrane helices (TMHMM) [24].

We compared HGT genes to those in the GenBank EST database to clarify the expression-pattern. We also searched the Taxonomically Broad EST eukaryotic database (TBestDB) [51] using TBLASTN with e-value 1e^−10^.

### Compositional Analysis

GC content, GC3s content, and codon usage of each gene in *P. teres*, *P. tritici-repentis*, and species with highest identity to HGT genes in non-fungi groups were calculated using CodonW (http://codonw.sourceforge.net/). Four indices of codon usage bias included CAI (codon adaptation index), CBI (codon bias index), FOP (frequency of optimal codon), and ENC (effective number of codons).

### Selection Analyses

We conducted selection pressure analyses for each HGT gene. Using Codeml in PAML 4 [52], we computed nonsynonymous (Ka) and synonymous (Ks) substitution rates, and then their ratio Omega (Ka/Ks). Omega values for the HGT genes were estimated both in fungal recipients and in their corresponding donor groups, bacteria or plants. We also used Codeml to detect probable positively selected sites and branches for these genes. In Codeml, the branch models allowed independent omega values for each branch. We estimated variation in Omega across the phylogeny, especially in lineages where transfer occurred [53]. Site-specific and branch-site models were used to identify potential positively selected codon sites and branches [52]. Evolutionary rates for HGT genes were compared using Omega values of HGT genes estimated in recipient species of *Pyrenophora* and in their corresponding donor groups [54].

## Supporting Information

Figure S1 **The phylogenetic trees of 16 types HGT genes in** ***Pyrenophora species*** **.** Bayesian trees are shown; the ML trees and NJ trees exhibited substantially the same topologies. Nodal support values ≥50 shown (BI/ML/NJ). Asterisks (*) indicate support values <50. *Pyrenophora* sequences are indicated in red, while HGT gene sequences from other fungi are indicated in green.
(PDF)

Figure S2 **Multiple alignment of amino acid sequences of enterochelin esterase-like enzyme in** ***Pyrenophora*** **.**
(DOC)

Figure S3 **GC and GC3s content of horizontally transferred genes in** ***P. tritici-repentis*** **and top-hit species in non-fungal groups.** GC3s and GC content of *P. tritici-repentis* are the mean values of the complete coding sequence (CDS). Gene 1–14 refers to the genes coding leucine-rich repeat protein, methyltransferase MppJ, beta-galactosidase, UDP-glucosyltransferase, GCN5-related N-acetyltransferase, oxidoreductase, Gfo/Idh/MocA family, enterochelin esterase-like enzyme, N-acetylglucosaminyltransferase, succinylglutamate desuccinylase/aspartoacylase, 5-formyltetrahydrofolate cyclo-ligase, NmrA family protein, glcG protein, xylanase A, cyanophycinase, respectively.
(TIF)

Figure S4 **The value of 4 index of codon bias: CAI, CBI, Fop and ENC of HGT genes,** ***P. tritici-repentis*** **and top-hit species in non-fungal groups.** CAI value. (B) CBI value. (C) Fop value. (D) ENC value. The CAI, CBI, Fop and ENC value of *P. tritici-repentis* are the mean value of all the CDS. Gene 1–14 refers to the genes coding leucine rich repeat protein, methyltransferase MppJ, beta-galactosidase, UDP-glucosyltransferase, GCN5-related N-acetyltransferase, oxidoreductase, Gfo/Idh/MocA family, enterochelin esterase-like enzyme, N-acetylglucosaminyltransferase, succinylglutamate desuccinylase/aspartoacylase, 5-formyltetrahydrofolate cyclo-ligase, NmrA family protein, glcG protein, xylanase A, cyanophycinase, respectively.
(TIF)

Table S1 **The amino acid (AA) lengths and number of introns of candidate HGT genes in the genomes of** ***Pyrenophora teres*** **and** ***P. tritici-repentis*** **.**
(DOC)

Table S2 **The synteny of HGT genes in** ***Pyrenophora*** **and closely related species.** Upstream gene of HGT, HGT genes and downstream gene of HGT in *P. teres* were used to compare with *P. tritici-repentis* and other closely related fungal recipients. If homologs of these genes located with seriation in other fungi, this HGT gene can be regarded as good synteny in recipients.
(XLS)

Table S3 **Lists of 93 fungi used in the analyses.** Species with red font means that they associate with plants in their lifestyles.
(XLS)
