# Supplementary material for: Multiple Interkingdom Horizontal Gene Transfers in Pyrenophora and Closely Related Species and Their Contributions to Phytopathogenic Lifestyles
Source: PLoS One. 2013 Mar 29;8(3):e60029. doi: 10.1371/journal.pone.0060029 (PMC3612039; doi:10.1371/journal.pone.0060029)
Supplement: Figure S1 — The phylogenetic trees of 16 types HGT genes in Pyrenophora species . Bayesian trees are shown; the ML trees and NJ trees exhibited substantially the same topologies. Nodal support values ≥50 shown (BI/ML/NJ). Asterisks (*) indicate support values <50. Pyrenophora sequences are indicated in red, while HGT gene sequences from other fungi are indicated in green. (PDF) [file pone.0060029.s001.pdf]

leucine rich repeat protein

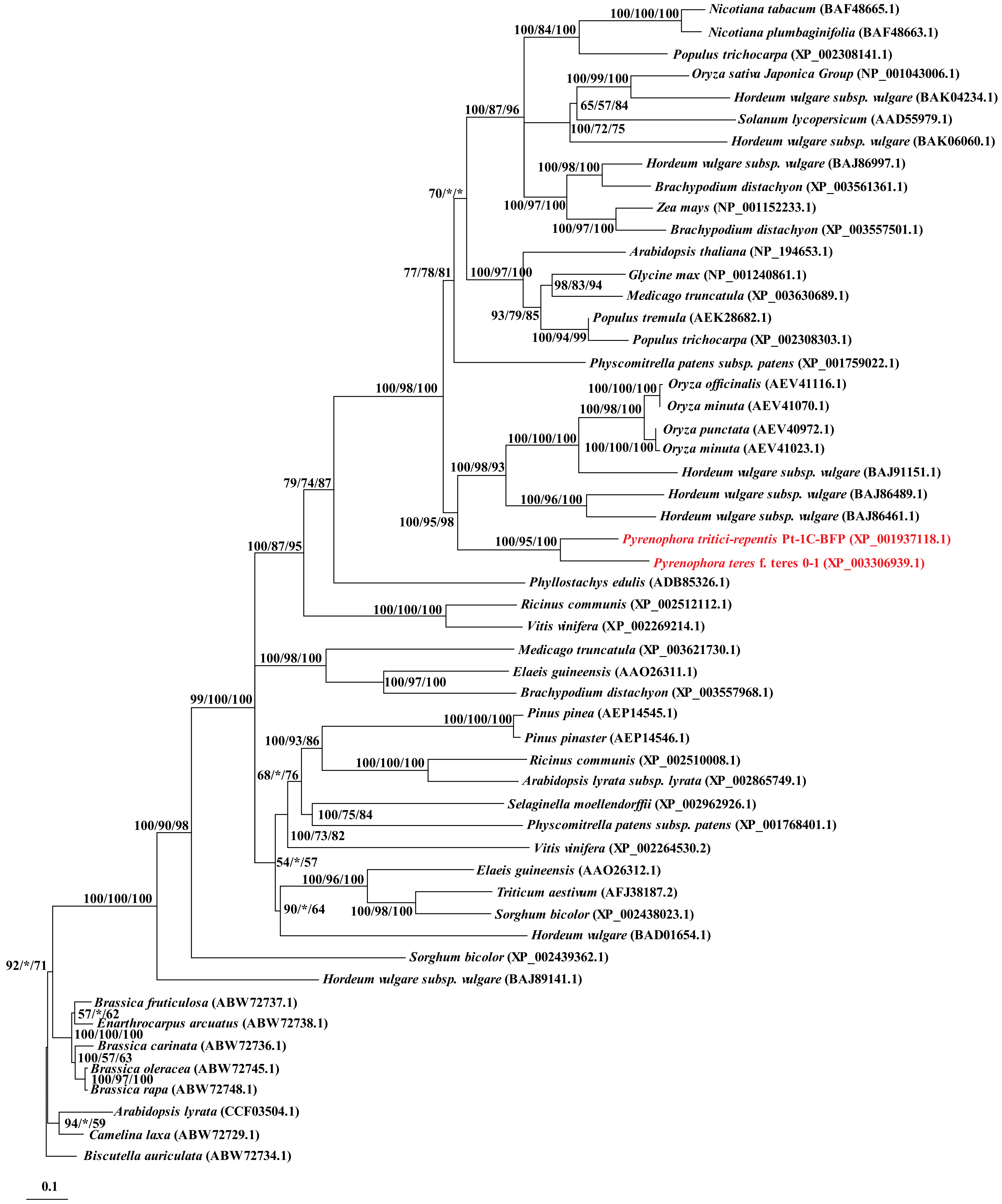

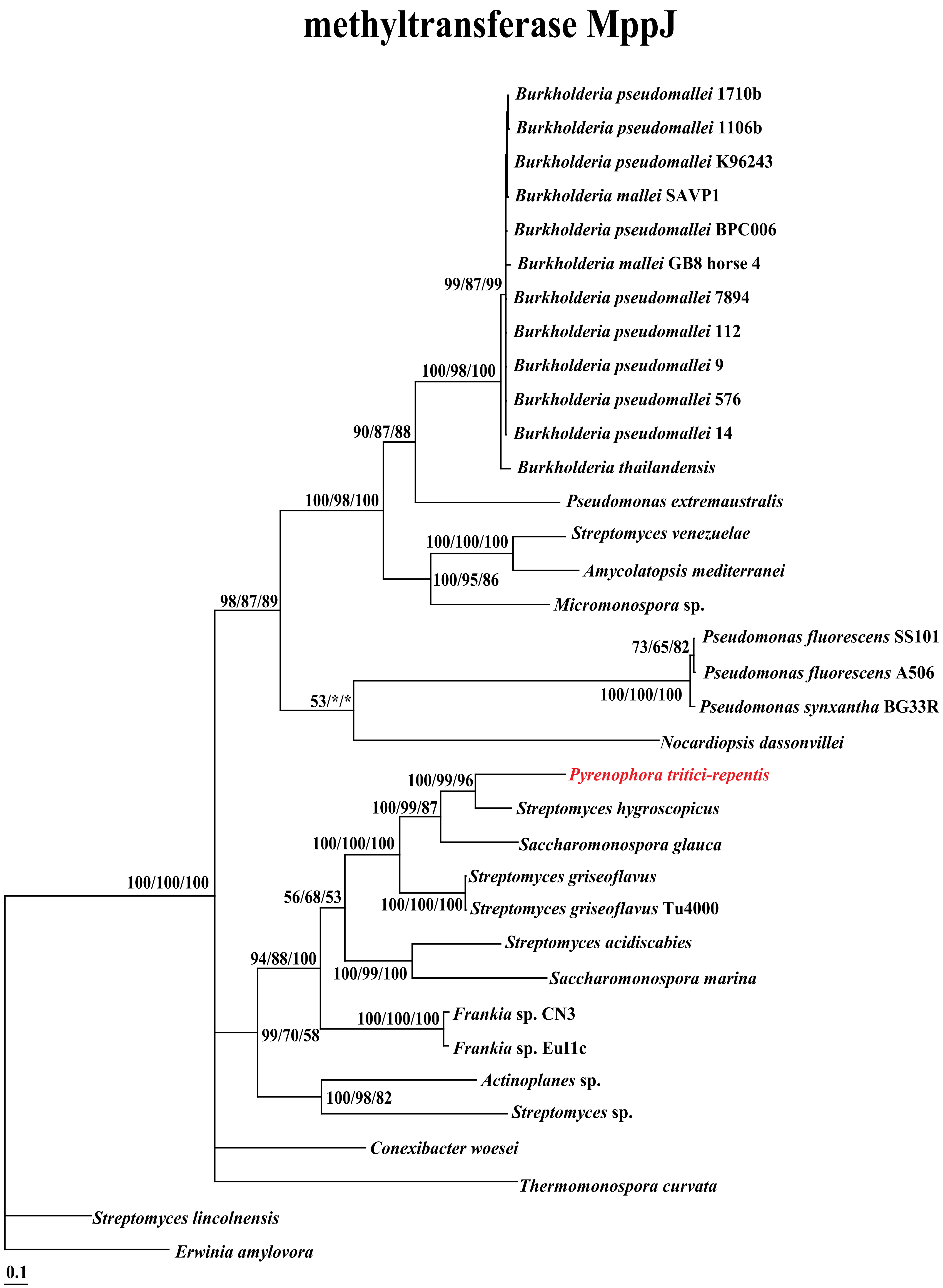
methyltransferase MppJ
Burkholderia pseudomallei 1710b
Burkholderia pseudomallei 1106b
Burkholderia pseudomallei K96243
Burkholderia mallei SAVP1
Burkholderia pseudomallei BPC006
Burkholderia mallei GB8 horse 4
99/87/99
Burkholderia pseudomallei 7894
Burkholderia pseudomallei 112
Burkholderia pseudomallei 9
Burkholderia pseudomallei 576
Burkholderia pseudomallei 14
Burkholderia thailandensis
100/98/100
90/87/88
Pseudomonas extremaustralis
100/98/100
Streptomyces venezuelae
100/100/100
Amycolatopsis mediterranei
100/95/86
Micromonospora sp.
98/87/89
Pseudomonas fluorescens SS101
73/65/82
Pseudomonas fluorescens A506
100/100/100
Pseudomonas synxantha BG33R
53/*/*
Nocardiopsis dassonvillei
Pyrenophora tritici-repentis
100/99/96
Streptomyces hygroscopicus
100/99/87
Saccharomonospora glauca
100/100/100
Streptomyces griseoflavus
Streptomyces griseoflavus Tu4000
100/100/100
100/100/100
56/68/53
Streptomyces acidiscabies
100/99/100
Saccharomonospora marina
94/88/100
Frankia sp. CN3
100/100/100
Frankia sp. EuI1c
99/70/58
Actinoplanes sp.
100/98/82
Streptomyces sp.
Conexibacter woesei
Thermomonospora curvata
Streptomyces lincolnensis
Erwinia amylovora
0.1

beta-galactosidase

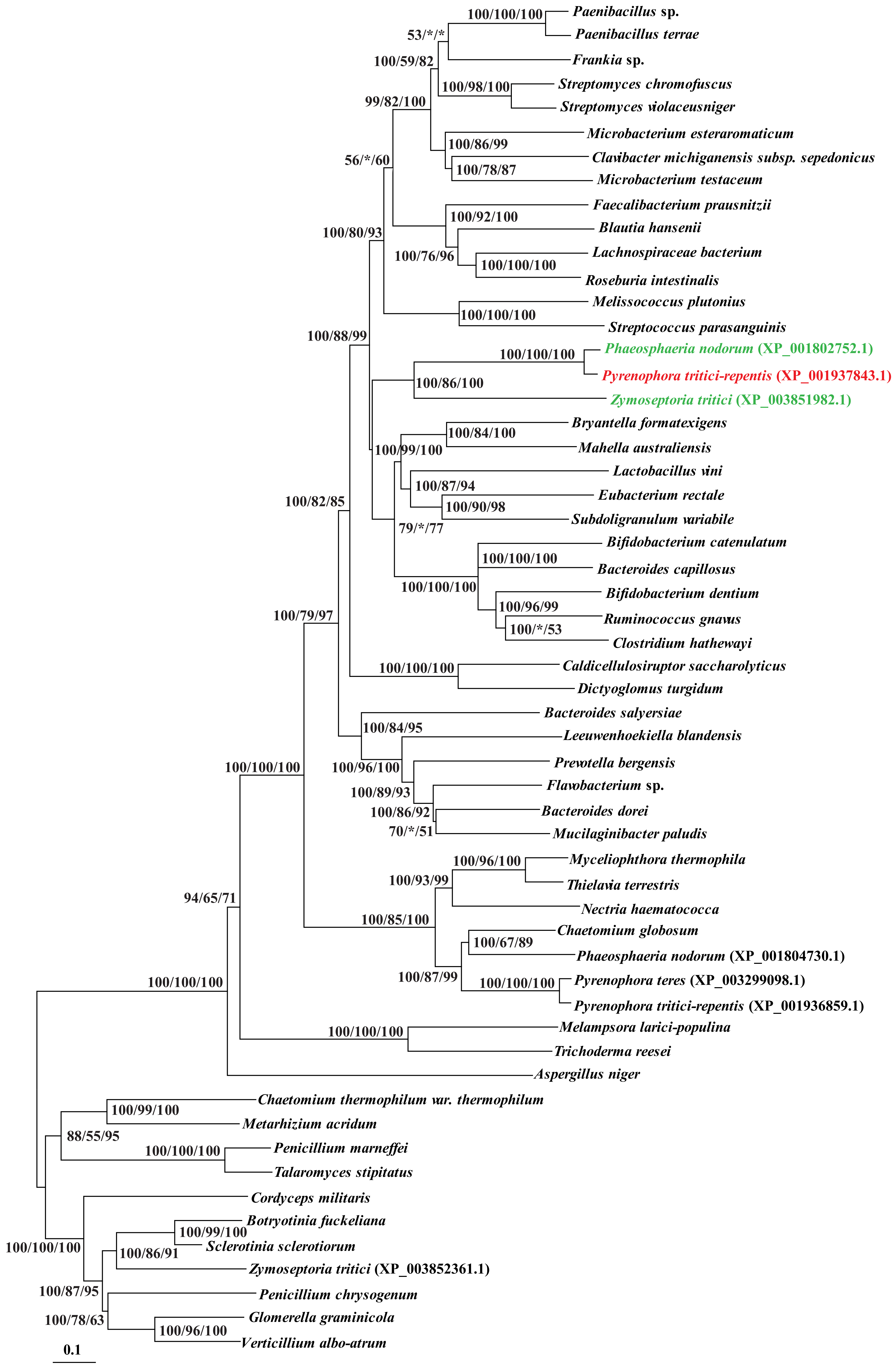

UDP-glucosyltransferase

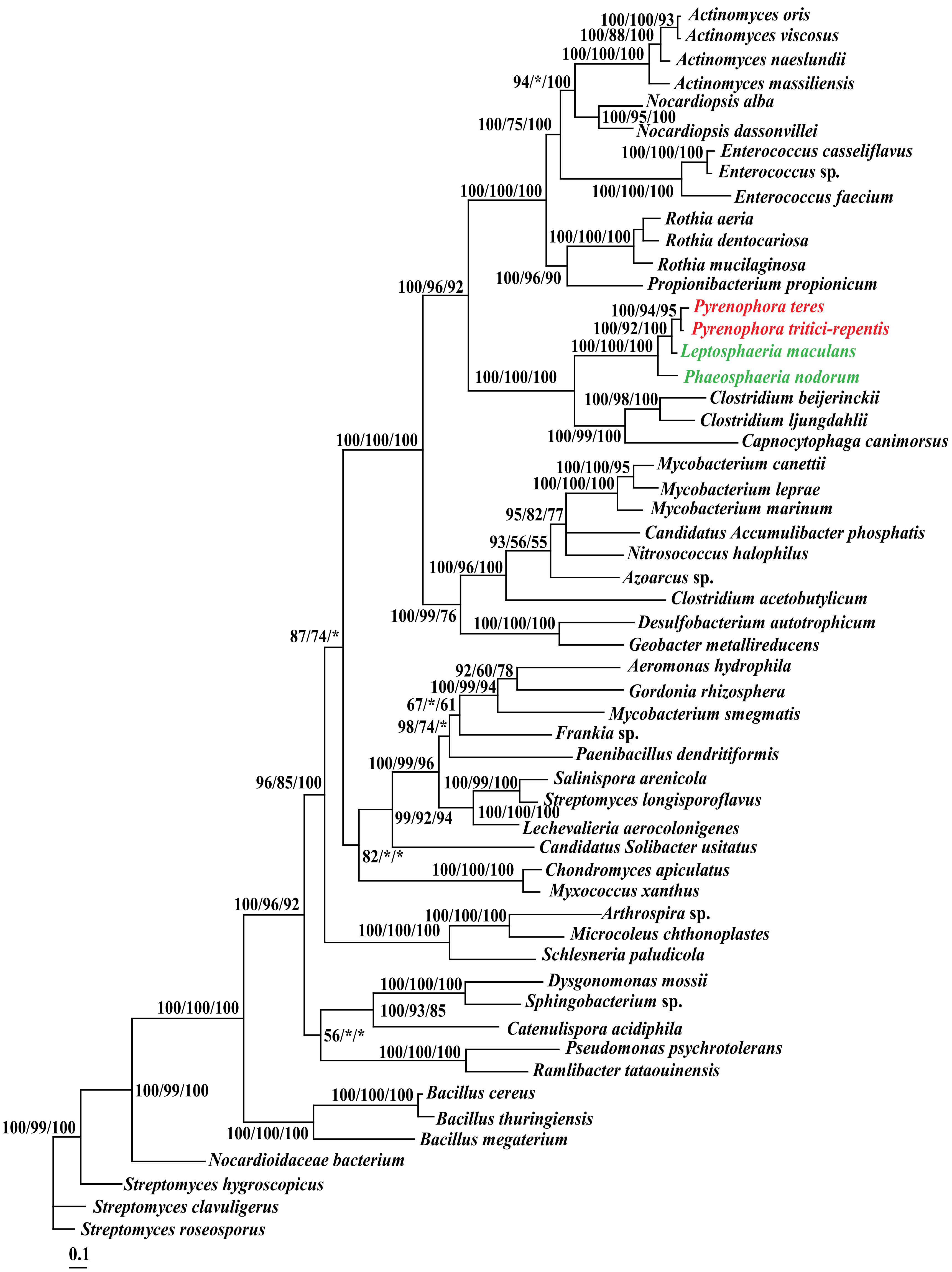

GCN5-related N-acetyltransferase

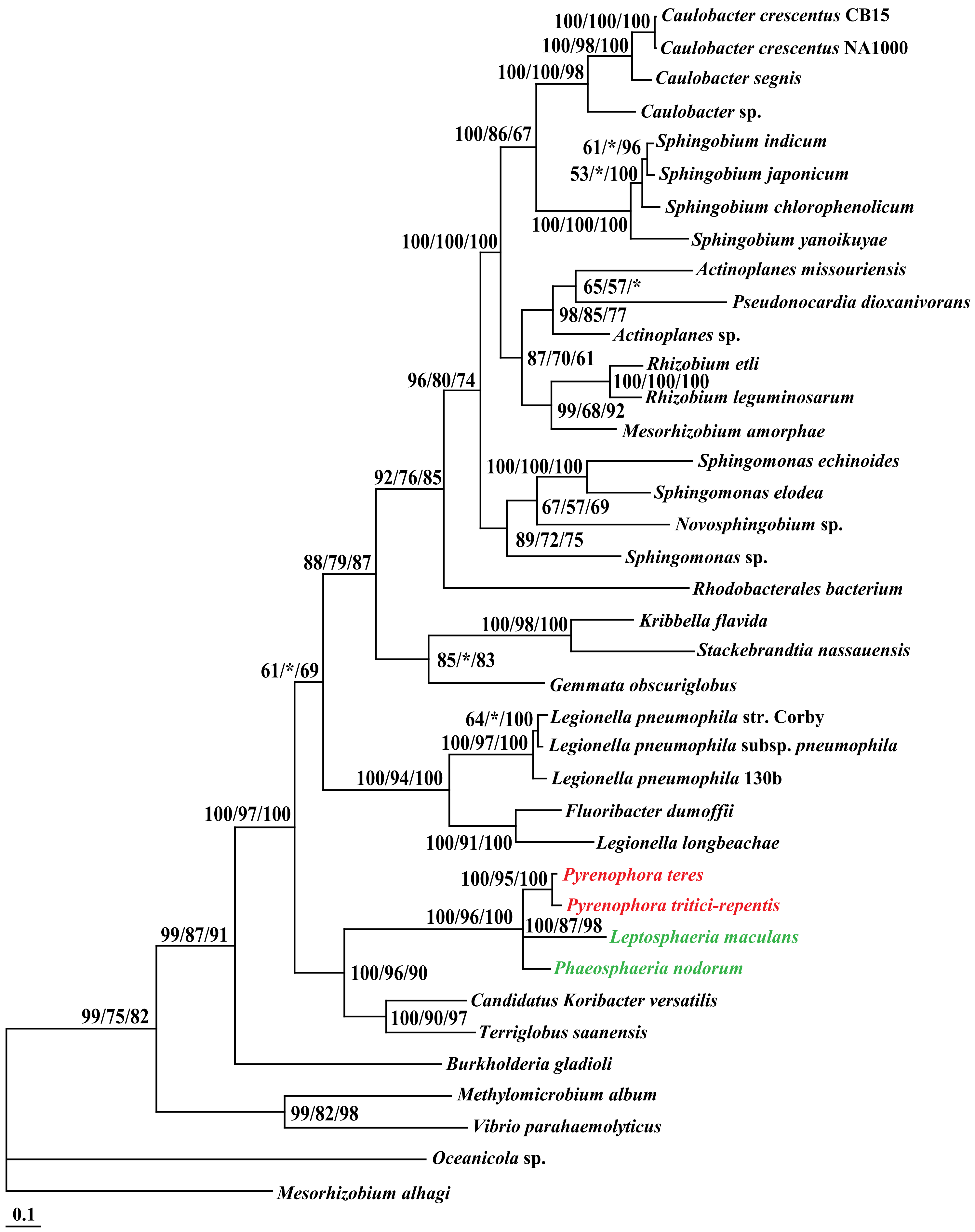

oxidoreductase, Gfo/Idh/MocA family

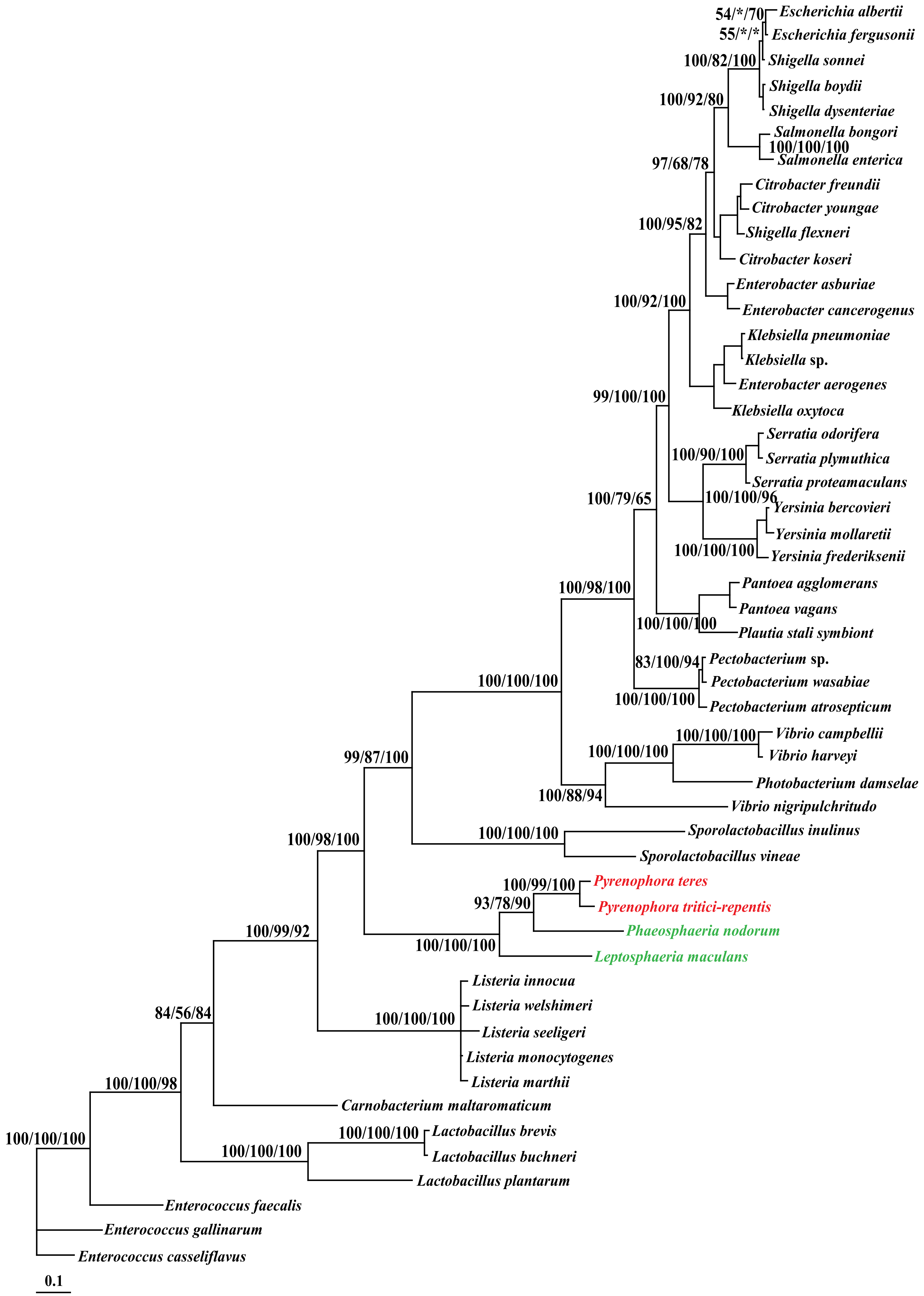

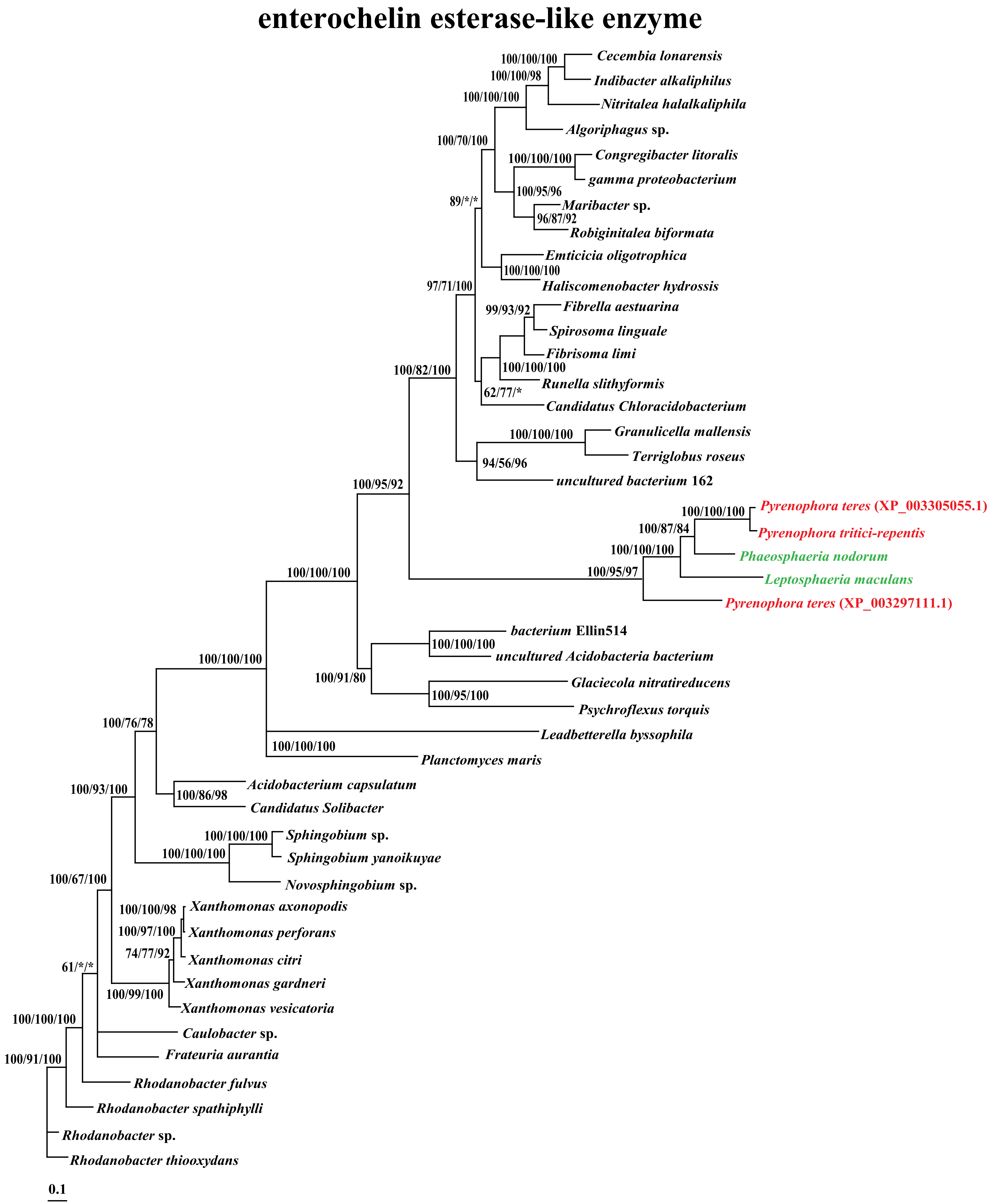

enterocheline esterase-like enzyme
Cecembia lonarensis
Indibacter alkaliphilus
Nitritalea halalkaliphila
Algoriphagus sp.
Congregibacter litoralis
gamma proteobacterium
Maribacter sp.
Robiginitalea biformata
Emticicia oligotrophica
Haliscomenobacter hydrossis
Fibrella aestuarina
Spirosoma linguale
Fibrisoma limi
Runella slithyformis
Candidatus Chloracidobacterium
Granulicella mallensis
Terriglobus roseus
uncultured bacterium 162
Pyrenophora teres (XP_003305055.1)
Pyrenophora tritici-repentis
Phaeosphaeria nodorum
Leptosphaeria maculans
Pyrenophora teres (XP_003297111.1)
bacterium Ellin514
uncultured Acidobacteria bacterium
Glaciecola nitratireducens
Psychroflexus torquis
Leadbetterella byssophila
Planctomyces maris
Acidobacterium capsulatum
Candidatus Solibacter
Sphingobium sp.
Sphingobium yanoikuyae
Novosphingobium sp.
Xanthomonas axonopodis
Xanthomonas perforans
Xanthomonas citri
Xanthomonas gardneri
Xanthomonas vesicatoria
Caulobacter sp.
Frateuria aurantia
Rhodanobacter fulvus
Rhodanobacter spathiphylli
Rhodanobacter sp.
Rhodanobacter thiooxydans
100/100/100
100/100/98
100/100/100
100/70/100
100/100/100
100/95/96
96/87/92
89/*/*
100/100/100
97/71/100
99/93/92
100/100/100
100/82/100
62/77/*
100/100/100
94/56/96
100/95/92
100/100/100
100/87/84
100/100/100
100/95/97
100/100/100
100/100/100
100/91/80
100/95/100
100/100/100
100/100/100
100/76/78
100/86/98
100/93/100
100/100/100
100/100/100
100/67/100
100/100/98
100/97/100
74/77/92
61/*/*
100/99/100
100/100/100
100/91/100
0.1

N-acetylglucosaminyltransferase

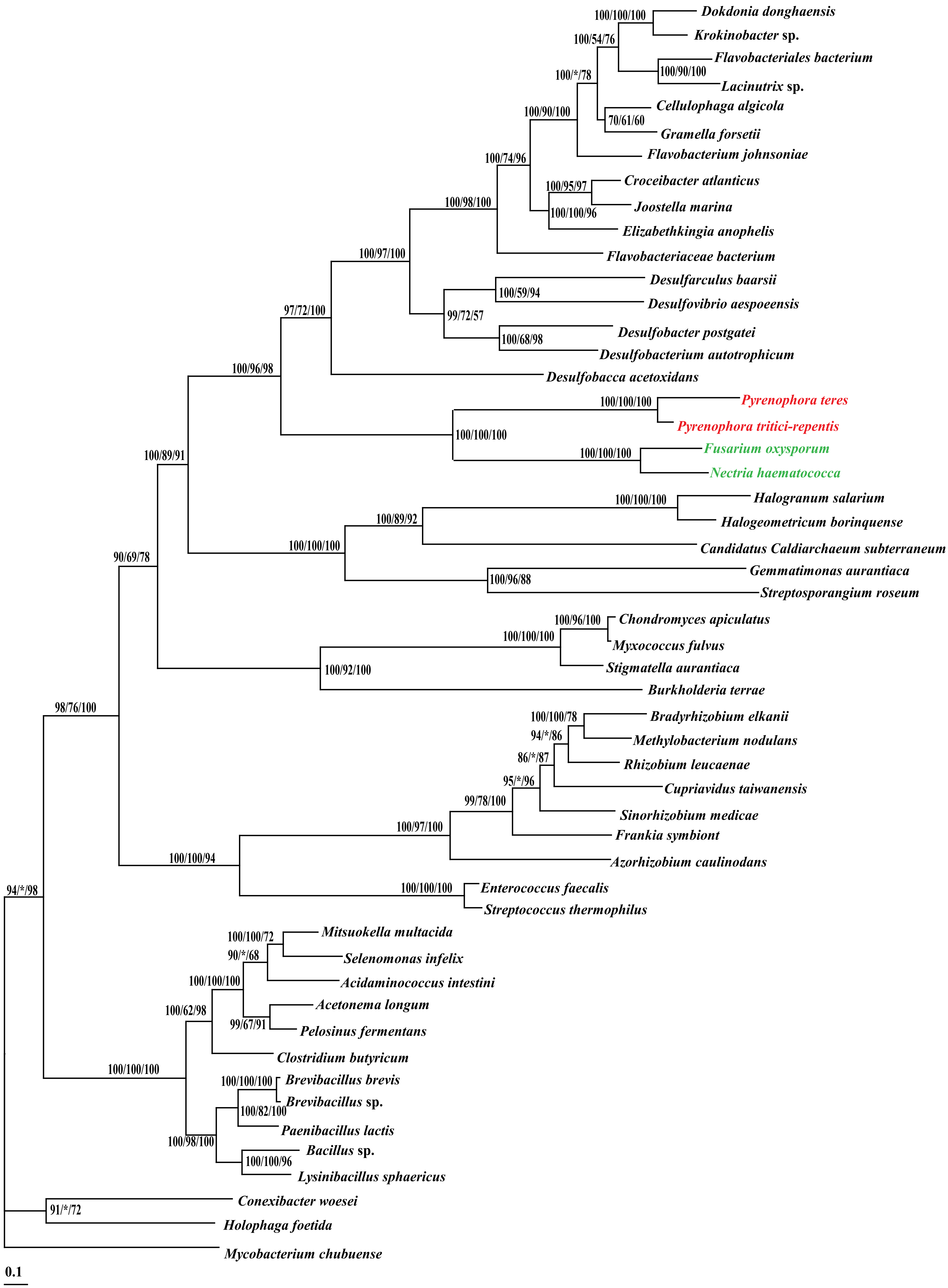

succinylglutamate desuccinylase/aspartoacylase

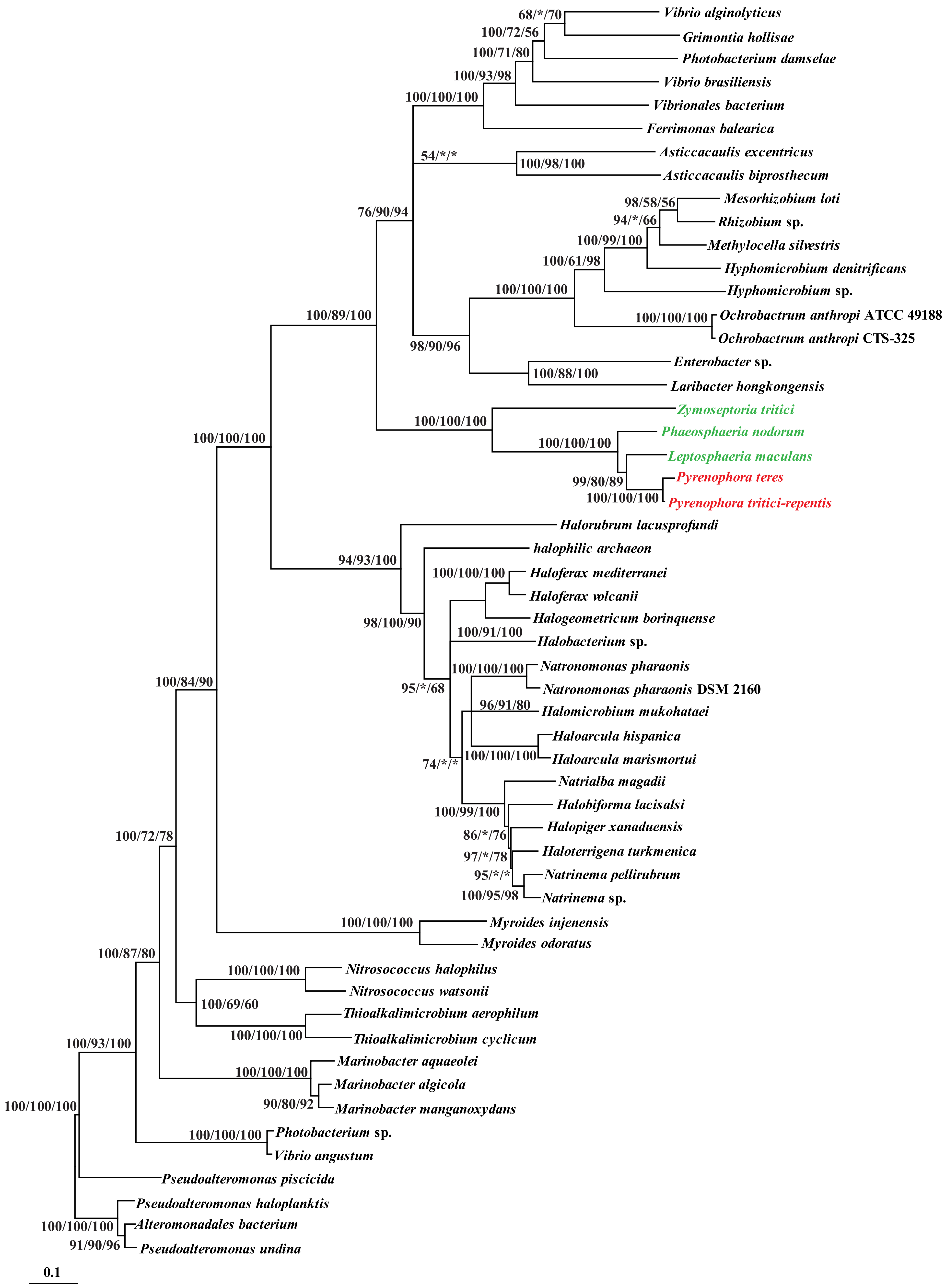

5-formyltetrahydrofolate cyclo-ligase

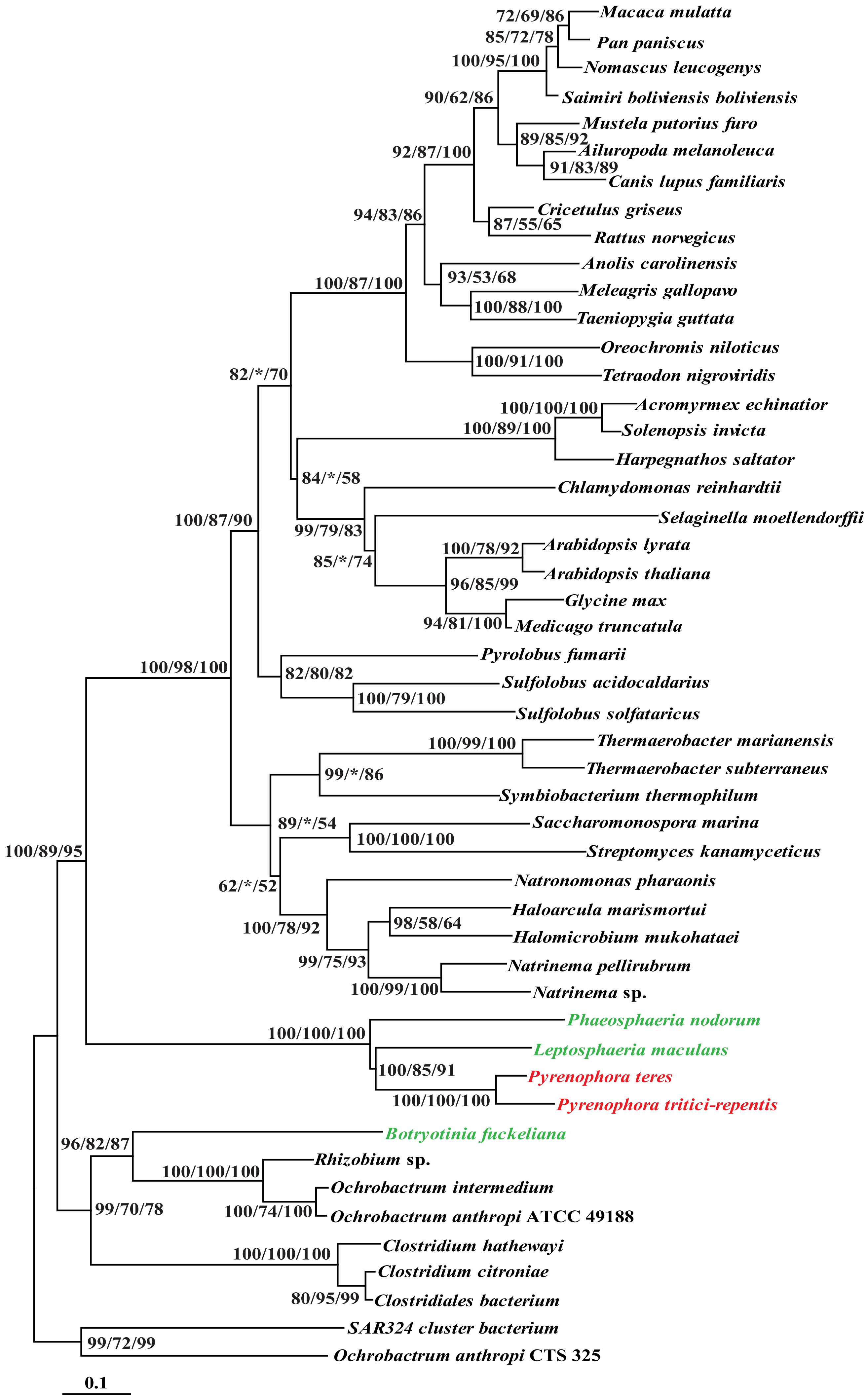

NmrA family protein

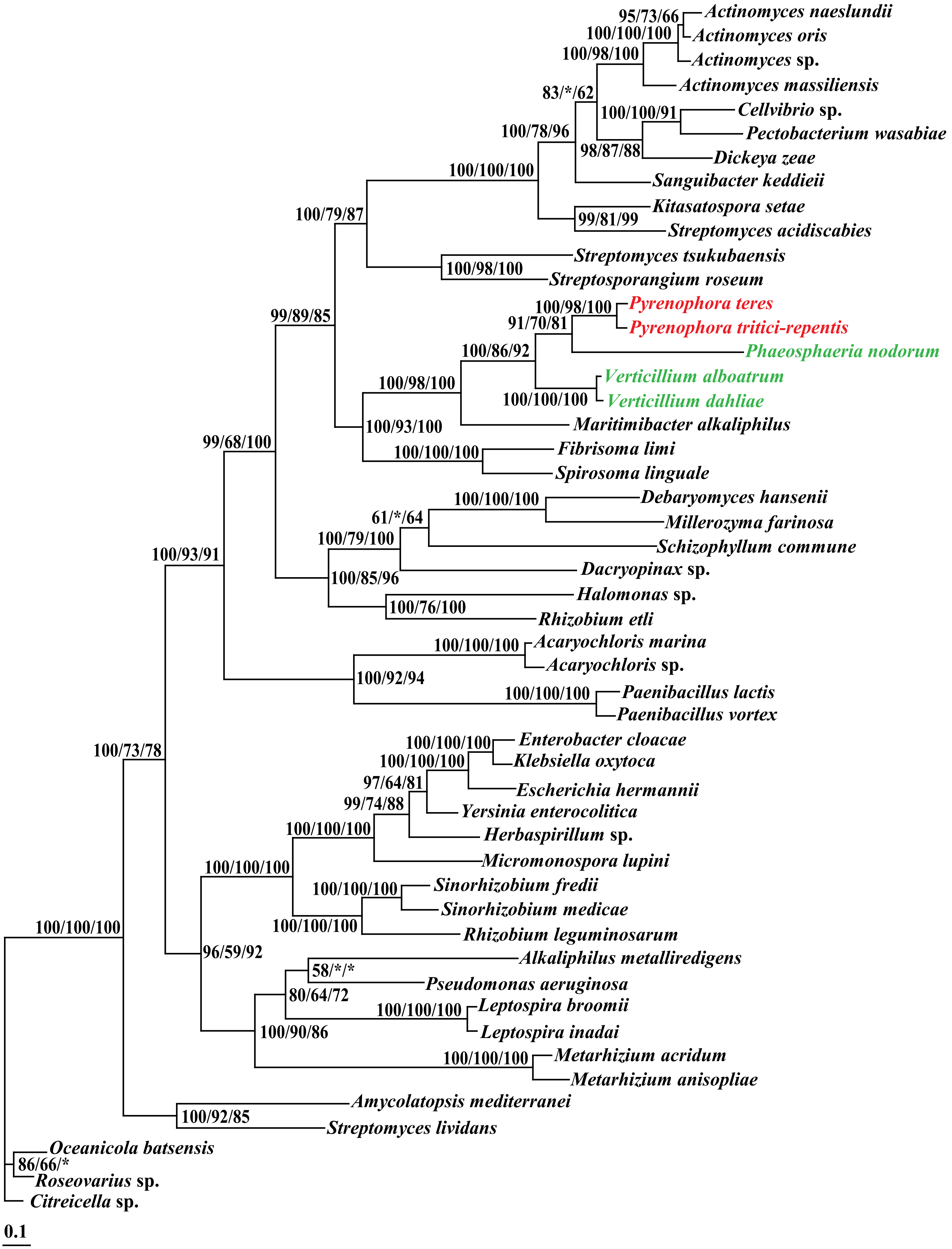

glcG protein

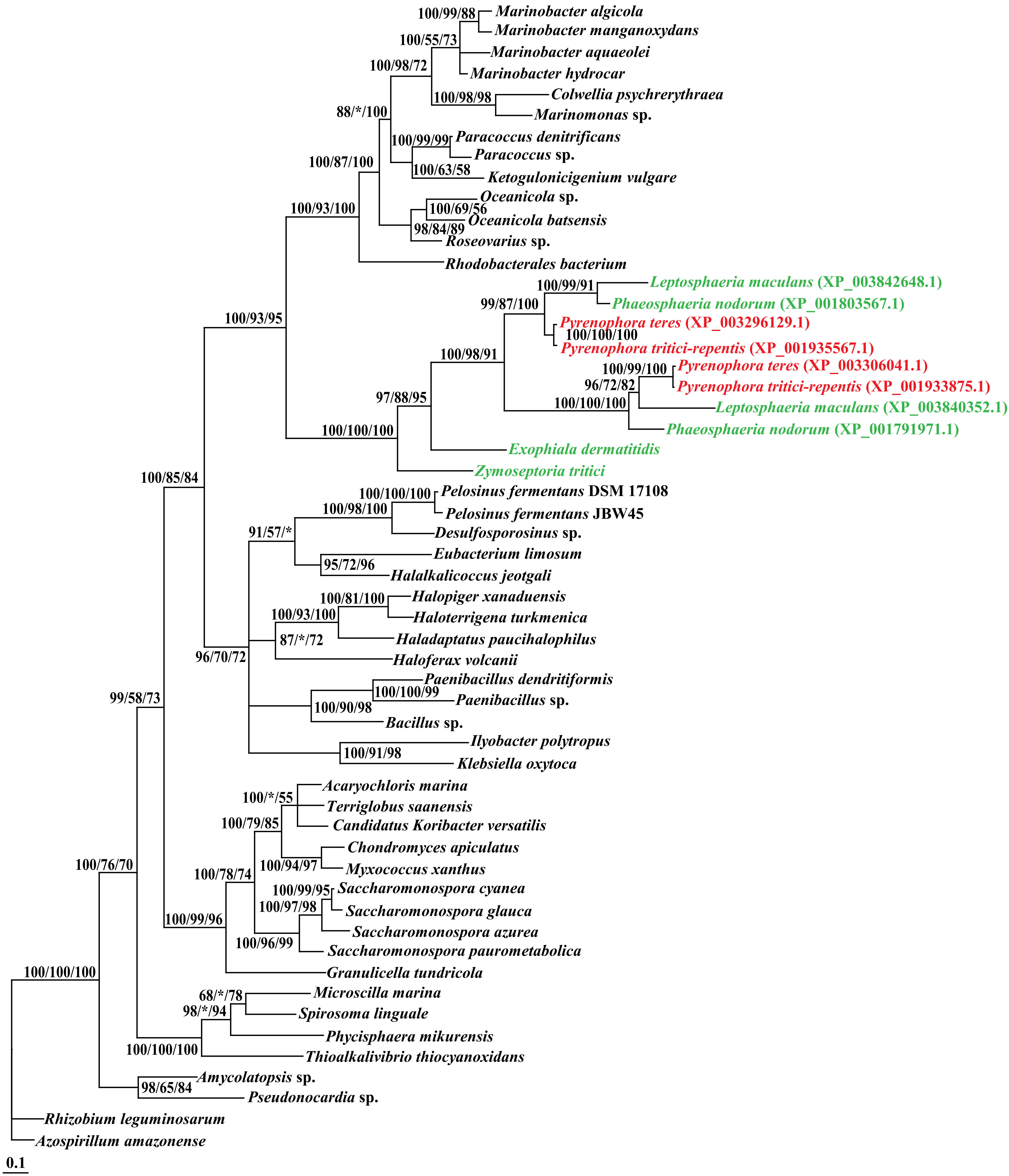

xylanase A

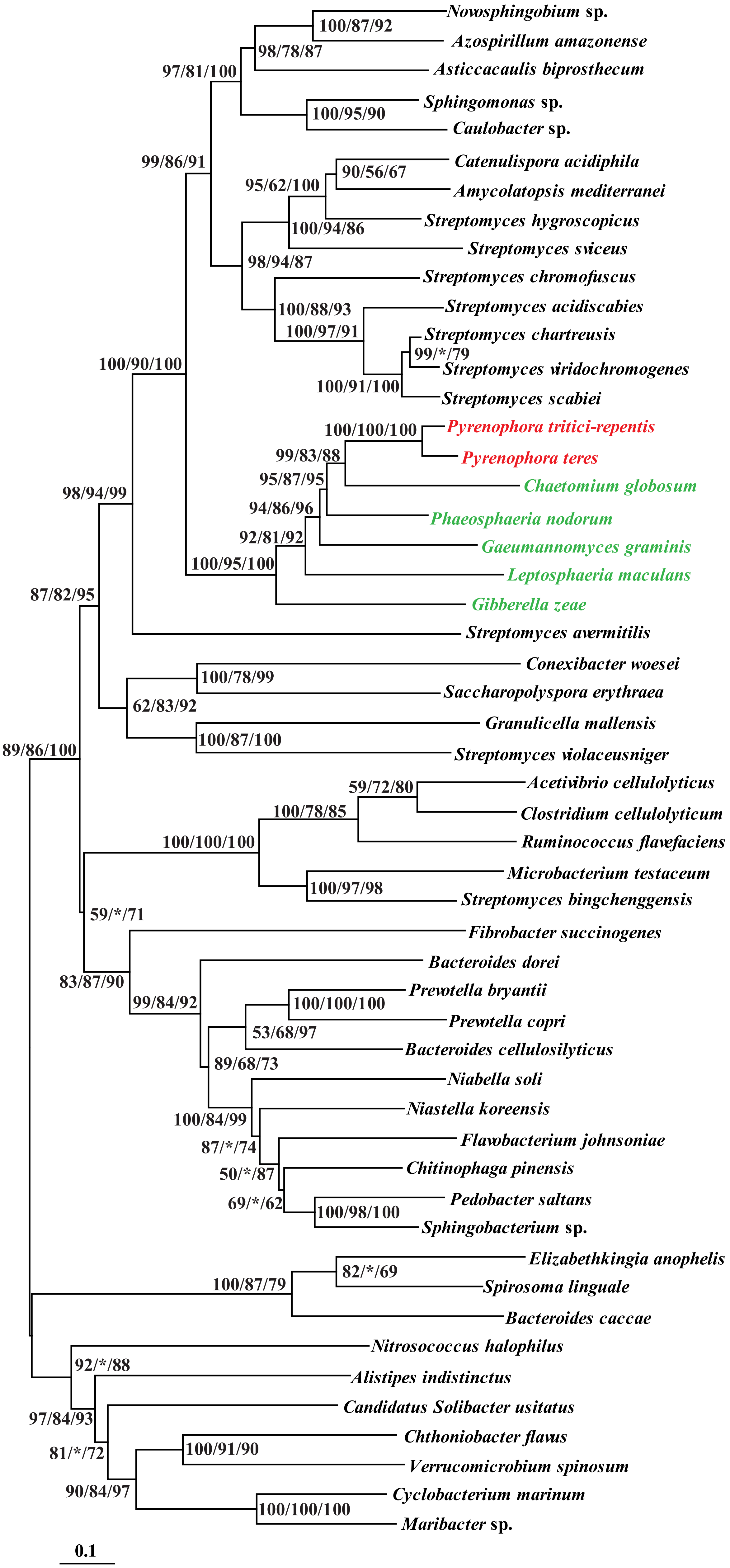
Nowosphingobium sp.
Azospirillum amazonense
Asticcacaulis biprosthecum
Sphingomonas sp.
Caulobacter sp.
Catenulispora acidiphila
Amycolatopsis mediterranei
Streptomyces hygroscopicus
Streptomyces sviceus
Streptomyces chromofuscus
Streptomyces acidiscabies
Streptomyces chartreusis
Streptomyces viridochromogenes
Streptomyces scabiei
Pyrenophora tritici-repentis
Pyrenophora teres
Chaetomium globosum
Phaeosphaeria nodorum
Gaeumannomyces graminis
Leptosphaeria maculans
Gibberella zeae
Streptomyces avermitilis
Conexibacter woesei
Saccharopolyspora erythraea
Granulicella mallensis
Streptomyces violaceusniger
Acetivibrio cellulolyticus
Clostridium cellulolyticum
Ruminococcus flavefaciens
Microbacterium testaceum
Streptomyces bingchenggensis
Fibrobacter succinogenes
Bacteroides dorei
Prevotella bryantii
Prevotella copri
Bacteroides cellulosilyticus
Niabella soli
Niastella koreensis
Flavobacterium johnsoniae
Chitinophaga pinensis
Pedobacter saltans
Sphingobacterium sp.
Elizabethkingia anophelis
Spirosoma linguale
Bacteroides caccae
Nitrosococcus halophilus
Alistipes indistinctus
Candidatus Solibacter usitatus
Chthoniobacter flavus
Verrucomicrobium spinosum
Cyclobacterium marinum
Maribacter sp.
100/87/92
98/78/87
97/81/100
100/95/90
99/86/91
90/56/67
95/62/100
100/94/86
98/94/87
100/88/93
100/97/91
99/*/79
100/91/100
100/90/100
100/100/100
99/83/88
95/87/95
94/86/96
92/81/92
100/95/100
98/94/99
87/82/95
100/78/99
62/83/92
100/87/100
89/86/100
59/72/80
100/78/85
100/100/100
100/97/98
59/*/71
83/87/90
99/84/92
100/100/100
53/68/97
89/68/73
100/84/99
87/*/74
50/*/87
69/*/62
100/98/100
82/*/69
100/87/79
92/*/88
97/84/93
81/*/72
100/91/90
90/84/97
100/100/100
0.1

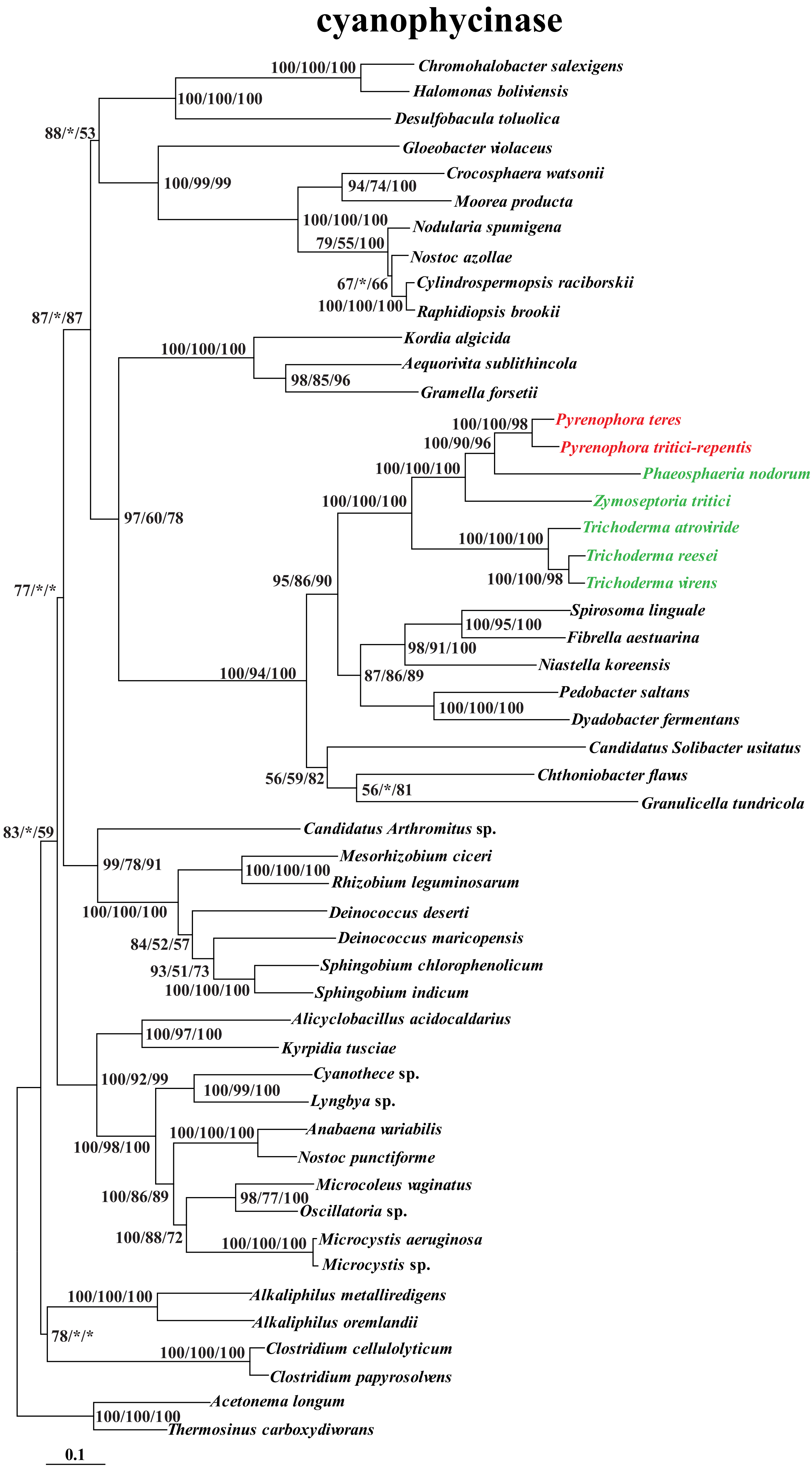

cyanophycinase
Chromohalobacter salexigens
Halomonas boliviensis
Desulfobacula toluolica
Gloeobacter violaceus
Crocosphaera watsonii
Moorea producta
Nodularia spumigena
Nostoc azollae
Cylindrospermopsis raciborskii
Raphidiopsis brookii
Kordia algicida
Aequorivita sublithincola
Gramella forsetii
Pyrenophora teres
Pyrenophora tritici-repentis
Phaeosphaeria nodorum
Zymoseptoria tritici
Trichoderma atroviride
Trichoderma reesei
Trichoderma virens
Spirosoma linguale
Fibrella aestuarina
Niastella koreensis
Pedobacter saltans
Dyadobacter fermentans
Candidatus Solibacter usitatus
Chthoniobacter flavus
Granulicella tundricola
Candidatus Arthromitus sp.
Mesorhizobium ciceri
Rhizobium leguminosarum
Deinococcus deserti
Deinococcus maricopensis
Sphingobium chlorophenolicum
Sphingobium indicum
Alicyclobacillus acidocaldarius
Kyrpidia tusciae
Cyanothece sp.
Lyngbya sp.
Anabaena variabilis
Nostoc punctiforme
Microcoleus vaginatus
Oscillatoria sp.
Microcystis aeruginosa
Microcystis sp.
Alkaliphilus metalliredigens
Alkaliphilus oremlandii
Clostridium cellulolyticum
Clostridium papyrosolvens
Acetonema longum
Thermosinus carboxydivorans
0.1

alpha/beta hydrolase

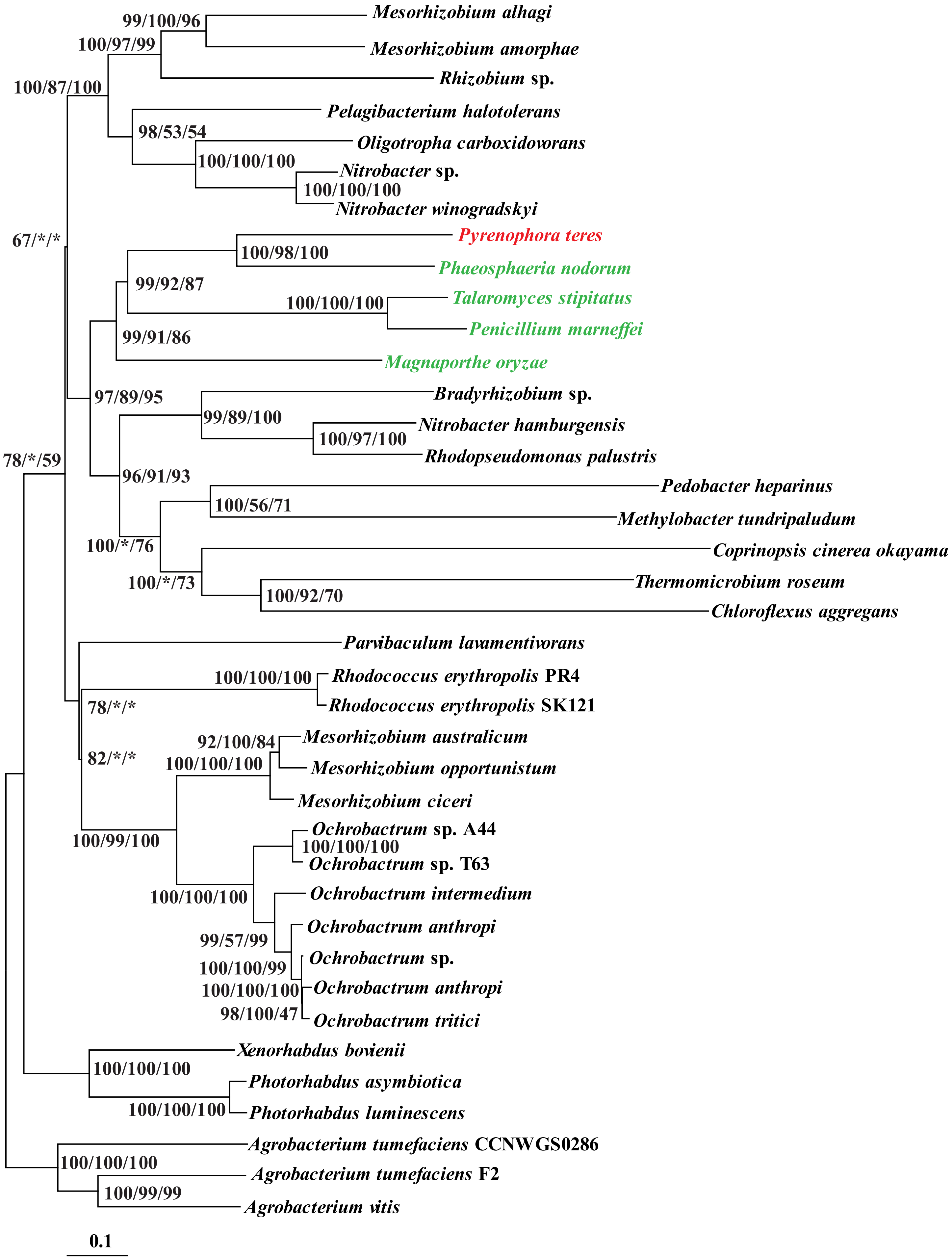

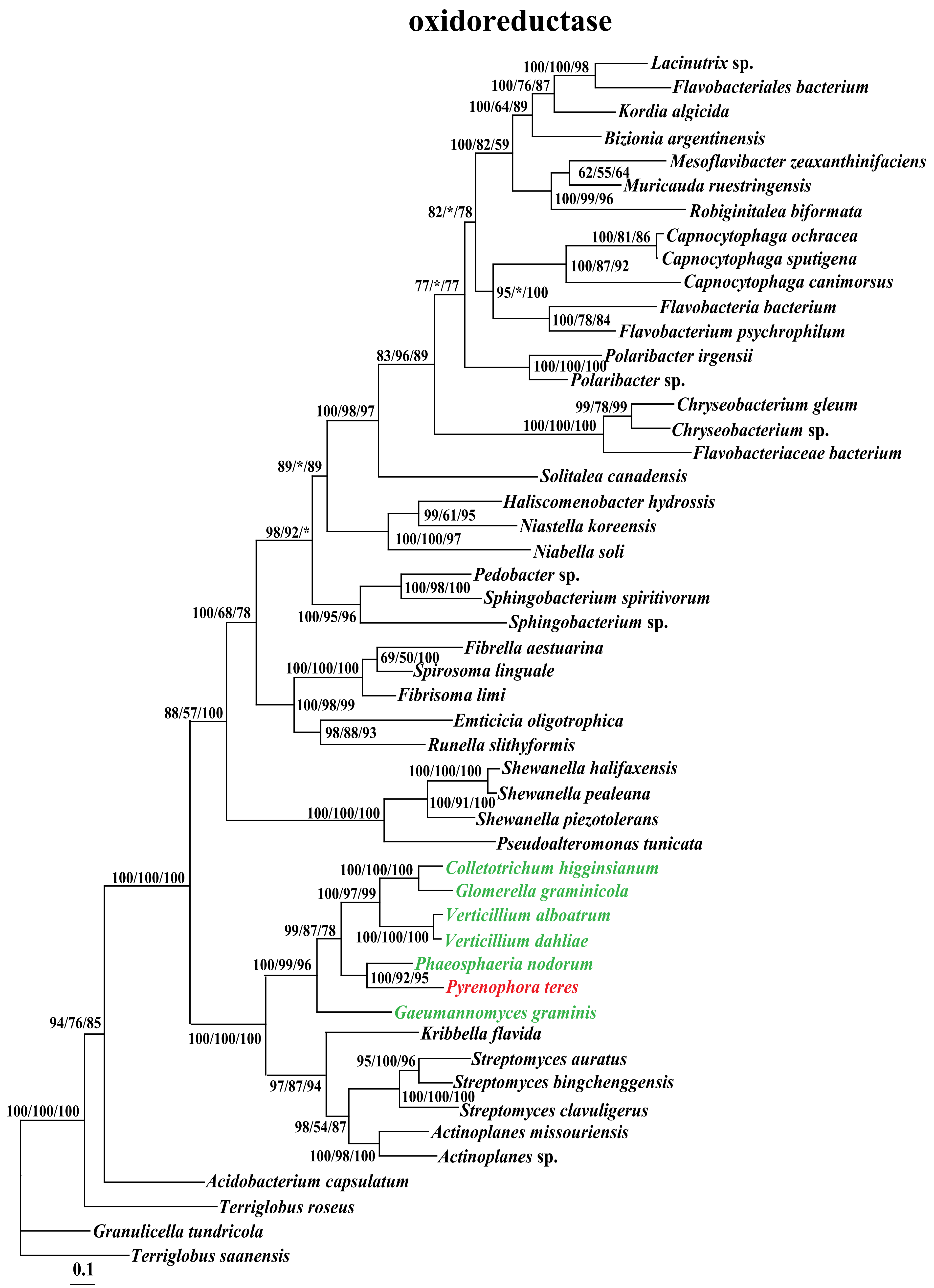

oxidoreductase
Lacinutrix sp.
Flavobacteriales bacterium
Kordia algicida
Bizionia argentinensis
Mesoflavibacter zeaxanthinifaciens
Muricauda ruestringensis
Robiginitalea biformata
Capnocytophaga ochracea
Capnocytophaga sputigena
Capnocytophaga canimorsus
Flavobacteria bacterium
Flavobacterium psychrophilum
Polaribacter irgensii
Polaribacter sp.
Chryseobacterium gleum
Chryseobacterium sp.
Flavobacteriaceae bacterium
Solitalea canadensis
Haliscomenobacter hydrossis
Niastella koreensis
Niabella soli
Pedobacter sp.
Sphingobacterium spiritivorum
Sphingobacterium sp.
Fibrella aestuarina
Spirosoma linguale
Fibrisoma limi
Emticicia oligotrophica
Runella slithyformis
Shewanella halifaxensis
Shewanella pealeana
Shewanella piezotolerans
Pseudoalteromonas tunicata
Colletotrichum higginsianum
Glomerella graminicola
Verticillium alboatrum
Verticillium dahliae
Phaeosphaeria nodorum
Pyrenophora teres
Gaeumannomyces graminis
Kribbella flavida
Streptomyces auratus
Streptomyces bingchenggensis
Streptomyces clavuligerus
Actinoplanes missouriensis
Actinoplanes sp.
Acidobacterium capsulatum
Terriglobus roseus
Granulicella tundricola
Terriglobus saanensis
100/100/98
100/76/87
100/64/89
100/82/59
62/55/64
100/99/96
82/*/78
100/81/86
100/87/92
95/*/100
77/*/77
100/78/84
83/96/89
100/100/100
99/78/99
100/98/97
100/100/100
89/*/89
99/61/95
98/92/*
100/100/97
100/98/100
100/68/78
100/95/96
69/50/100
100/100/100
100/98/99
88/57/100
98/88/93
100/100/100
100/91/100
100/100/100
100/100/100
100/100/100
100/97/99
99/87/78
100/100/100
100/99/96
100/92/95
94/76/85
100/100/100
95/100/96
97/87/94
100/100/100
98/54/87
100/100/100
100/98/100
0.1
