# Supplementary figures and images for: Multiple Interkingdom Horizontal Gene Transfers in Pyrenophora and Closely Related Species and Their Contributions to Phytopathogenic Lifestyles
Source: PLoS One. 2013 Mar 29;8(3):e60029. doi: 10.1371/journal.pone.0060029 (PMC3612039; doi:10.1371/journal.pone.0060029)

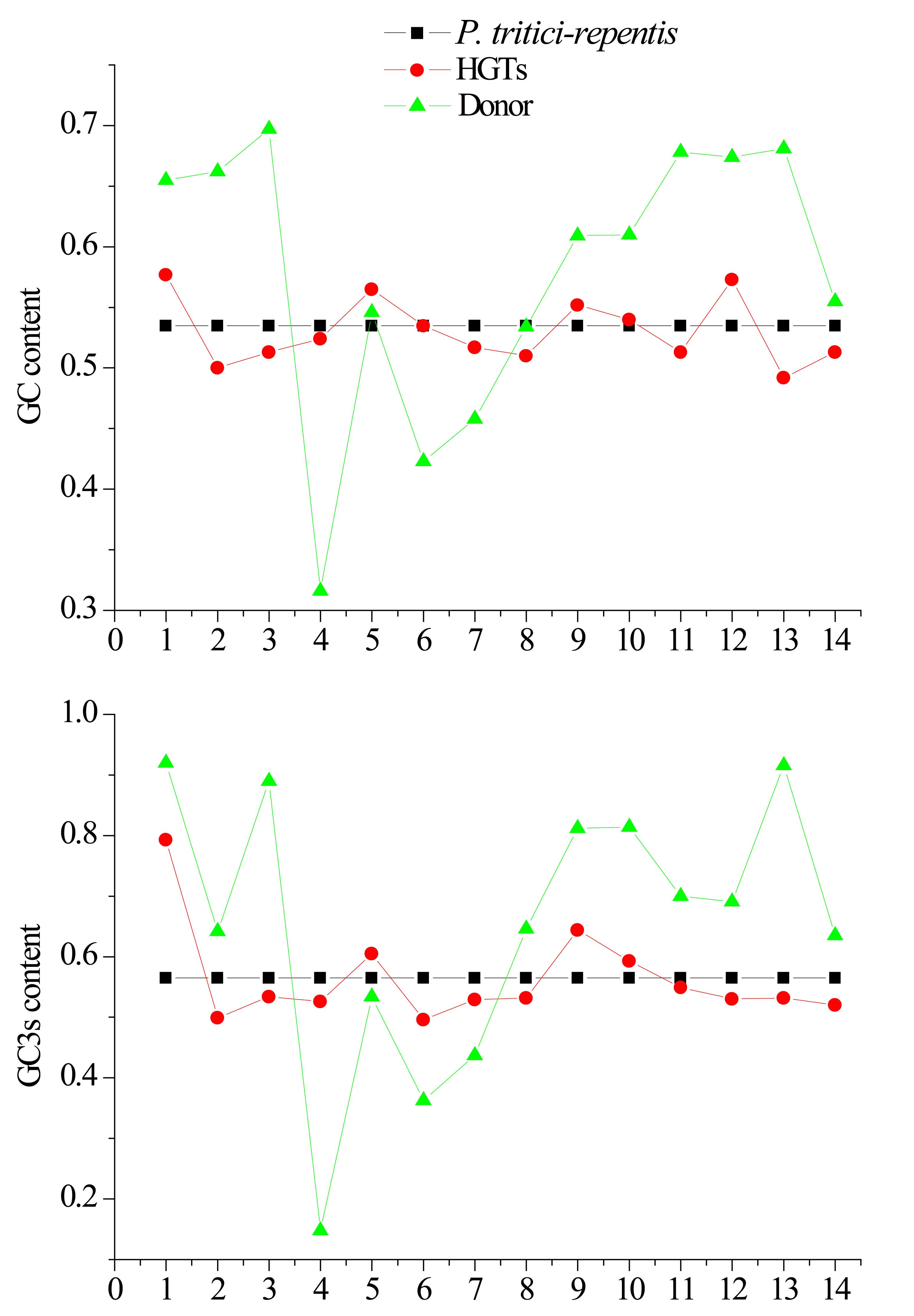

Supplement: Figure S3 — GC and GC3s content of horizontally transferred genes in P. tritici-repentis and top-hit species in non-fungal groups. GC3s and GC content of P. tritici-repentis are the mean values of the complete coding sequence (CDS). Gene 1–14 refers to the genes coding leucine-rich repeat protein, methyltransferase MppJ, beta-galactosidase, UDP-glucosyltransferase, GCN5-related N-acetyltransferase, oxidoreductase, Gfo/Idh/MocA family, enterochelin esterase-like enzyme, N-acetylglucosaminyltransferase, succinylglutamate desuccinylase/aspartoacylase, 5-formyltetrahydrofolate cyclo-ligase, NmrA family protein, glcG protein, xylanase A, cyanophycinase, respectively. (TIF) [file pone.0060029.s003.tif]

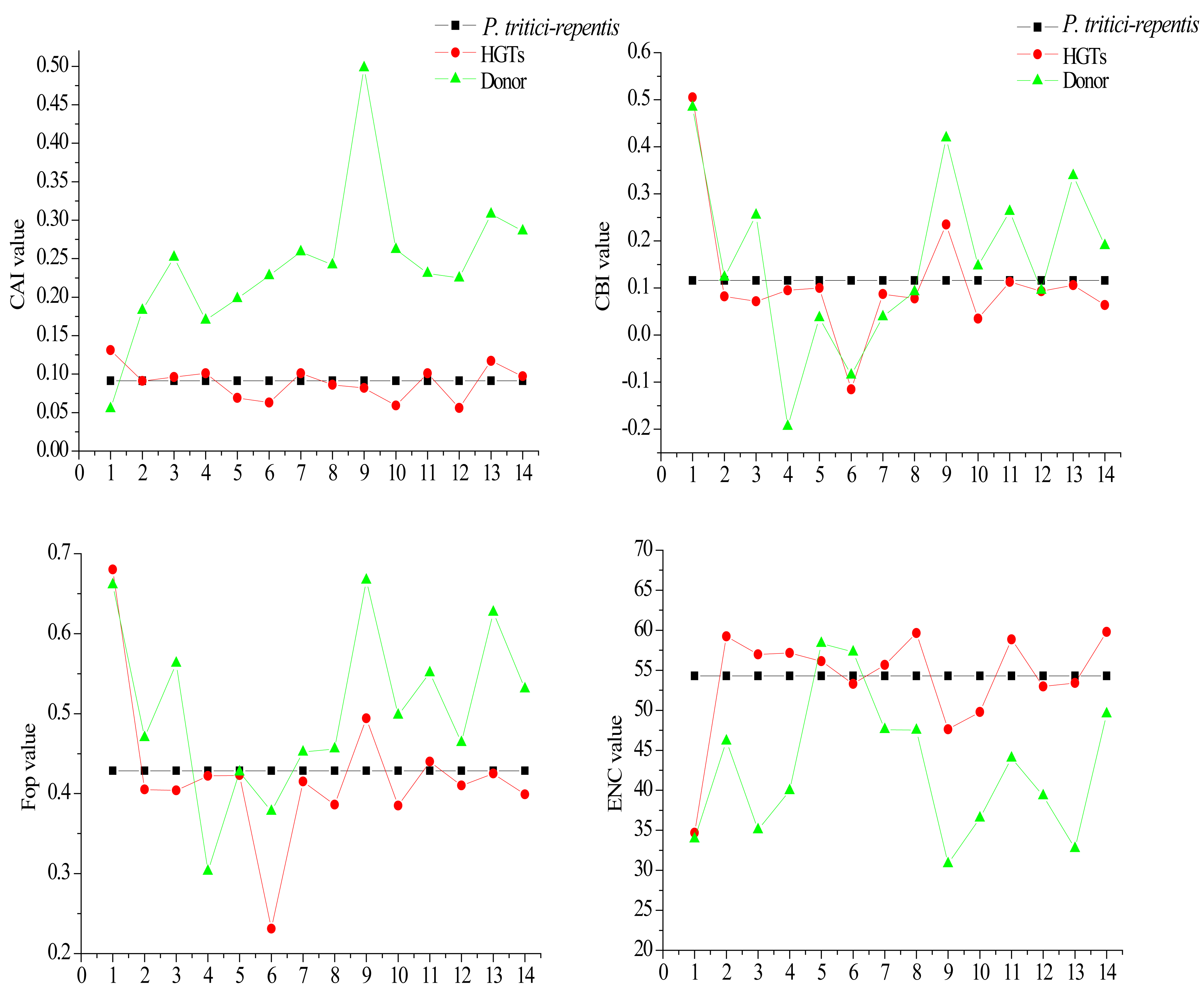

Supplement: Figure S4 — The value of 4 index of codon bias: CAI, CBI, Fop and ENC of HGT genes, P. tritici-repentis and top-hit species in non-fungal groups. CAI value. (B) CBI value. (C) Fop value. (D) ENC value. The CAI, CBI, Fop and ENC value of P. tritici-repentis are the mean value of all the CDS. Gene 1–14 refers to the genes coding leucine rich repeat protein, methyltransferase MppJ, beta-galactosidase, UDP-glucosyltransferase, GCN5-related N-acetyltransferase, oxidoreductase, Gfo/Idh/MocA family, enterochelin esterase-like enzyme, N-acetylglucosaminyltransferase, succinylglutamate desuccinylase/aspartoacylase, 5-formyltetrahydrofolate cyclo-ligase, NmrA family protein, glcG protein, xylanase A, cyanophycinase, respectively. (TIF) [file pone.0060029.s004.tif]
