# Supplementary material for: Multiple Interkingdom Horizontal Gene Transfers in Pyrenophora and Closely Related Species and Their Contributions to Phytopathogenic Lifestyles
Source: PLoS One. 2013 Mar 29;8(3):e60029. doi: 10.1371/journal.pone.0060029 (PMC3612039; doi:10.1371/journal.pone.0060029)
Supplement: Table S1 — The amino acid (AA) lengths and number of introns of candidate HGT genes in the genomes of Pyrenophora teres and P. tritici-repentis . (DOC) [file pone.0060029.s005.doc]

The amino acid (AA) lengths and number of introns of candidate HGT genes in the genomes of [*Pyrenophora teres*](http://www.ncbi.nlm.nih.gov/genome/2995) and [*P. tritici-repentis*](http://www.ncbi.nlm.nih.gov/genome/706)

|  | Gene name | Species | Gene ID | Amino acid lengths | Introns |
| --- | --- | --- | --- | --- | --- |
| 1 | leucine rich repeat protein | *P. teres* | XP_003306939.1 | 321 | 0 |
|  |  | [*P. tritici-repentis*](http://www.ncbi.nlm.nih.gov/genome/706) | XP_001937118.1 | 304 | 0 |
| 2 | methyltransferase MppJ | [*P. tritici-repentis*](http://www.ncbi.nlm.nih.gov/genome/706) | XP_001942153.1 | 340 | 0 |
| 3 | beta-galactosidase | [*P. tritici-repentis*](http://www.ncbi.nlm.nih.gov/genome/706) | XP_001937843.1 | 806 | 0 |
| 4 | UDP-glucosyltransferase | *P. teres* | XP_003299971.1 | 434 | 2 |
|  |  | [*P. tritici-repentis*](http://www.ncbi.nlm.nih.gov/genome/706) | XP_001934409.1 | 418 | 1 |
| 5 | GCN5-related N-acetyltransferase | *P. teres* | XP_003296325.1 | 215 | 1 |
|  |  | [*P. tritici-repentis*](http://www.ncbi.nlm.nih.gov/genome/706) | XP_001932301.1 | 209 | 1 |
| 6 | oxidoreductase, Gfo/Idh/MocA family | *P. teres* | XP_003304278.1 | 331 | 0 |
|  |  | [*P. tritici-repentis*](http://www.ncbi.nlm.nih.gov/genome/706) | XP_001940400.1 | 331 | 0 |
| 7 | enterochelin esterase-like enzyme | *P. teres* | XP_003305055.1 | 687 | 1 |
|  |  | *P. teres* | XP_003297111.1 | 472 | 0 |
|  |  | [*P. tritici-repentis*](http://www.ncbi.nlm.nih.gov/genome/706) | XP_001930965.1 | 661 | 2 |
| 8 | N-acetylglucosaminyltransferase | *P. teres* | XP_003300109.1 | 438 | 1 |
|  |  | [*P. tritici-repentis*](http://www.ncbi.nlm.nih.gov/genome/706) | XP_001938894.1 | 512 | 0 |
| 9 | succinylglutamate desuccinylase/aspartoacylase | *P. teres* | XP_003305149.1 | 382 | 2 |
|  |  | [*P. tritici-repentis*](http://www.ncbi.nlm.nih.gov/genome/706) | XP_001940578.1 | 370 | 3 |
| 10 | 5-formyltetrahydrofolate cyclo-ligase | *P. teres* | XP_003299132.1 | 304 | 0 |
|  |  | [*P. tritici-repentis*](http://www.ncbi.nlm.nih.gov/genome/706) | XP_001937221.1 | 189 | 0 |
| 11 | NmrA family protein | *P. teres* | XP_003299846.1 | 304 | 0 |
|  |  | [*P. tritici-repentis*](http://www.ncbi.nlm.nih.gov/genome/706) | XP_001935333.1 | 304 | 0 |
| 12 | glcG protein | *P. teres* | XP_003296129.1 | 147 | 0 |
|  |  | *P. teres* | XP_003306041.1 | 245 | 1 |
|  |  | [*P. tritici-repentis*](http://www.ncbi.nlm.nih.gov/genome/706) | XP_001935567.1 | 147 | 0 |
|  |  | [*P. tritici-repentis*](http://www.ncbi.nlm.nih.gov/genome/706) | XP_001933875.1 | 245 | 1 |
| 13 | xylanase A | *P. teres* | XP_003299438.1 | 447 | 2 |
|  |  | [*P. tritici-repentis*](http://www.ncbi.nlm.nih.gov/genome/706) | XP_001935182.1 | 631 | 1 |
| 14 | cyanophycinase | *P. teres* | XP_003304690.1 | 289 | 1 |
|  |  | [*P. tritici-repentis*](http://www.ncbi.nlm.nih.gov/genome/706) | XP_001938001.1 | 289 | 1 |
| 15 | alpha/beta hydrolase | *P. teres* | XP_003297280.1 | 325 | 0 |
| 16 | oxidoreductase | *P. teres* | XP_003305619.1 | 357 | 0 |
