# Supplementary material for: Multiple Interkingdom Horizontal Gene Transfers in Pyrenophora and Closely Related Species and Their Contributions to Phytopathogenic Lifestyles
Source: PLoS One. 2013 Mar 29;8(3):e60029. doi: 10.1371/journal.pone.0060029 (PMC3612039; doi:10.1371/journal.pone.0060029)
Supplement: Supplementary file 4 [file pone.0060029.s002.doc]

```
XP_003297111.1                                                                                                          0
XP_003305055.1    MLKAVILYATIALSATAVPTTWNHAERNTNLNIKLSVANGLLSSPTDGRIVLMFAPNGTDPLEDTDVSTSKNKIYGKNVYQFGPKTTVVFSGGGNEDTER    100
XP_001930965.1    MLKAAILYATIALSATAVPTTRNHAERNTDLDIKLSVANGLLSSPTDGRIVLIFAPNGTDPLEDTDVTTSKNKIYGKNVYQFGPKTAVVFSDGGSDDAES    100

XP_003297111.1                                                                                                          0
XP_003305055.1    GVFGWPNVSLSYVEPGTYNVQGFLTRYEKVTRSDGSTVSVRFPCGDGAPNVNGFGSLVTSVTKVVVSGGSQKLELTFNNVTVVEGLTGKEIGGCNQGNYA    200
XP_001930965.1    GVFGWPNVSLSDVEPGTYNVQGFLTRYERVTRSDGSIVNVRFPCVDGAPNVNGFGSLVTSVTKVVVSGGSQKLELTFNNVTTVDGLTGKEIGGCNQGNYA    200

XP_003297111.1                          MYIGAKVLLPAGYNASDTLTRYPVVYSQFHWLG..GESWWSHDAAFTEAWHNGMIPAQDGKPARPTPKLIIVMFRHEN     76
XP_003305055.1    DTERLKYVKIRSKKLSKFWGRDMFVGANIVLPAGYNN...QTRYPVIYHQNHWAGSDGAYGYGTNEKFTVAWDAGEIPATNRTAARPAPKFIMVSFRHES    297
XP_001930965.1    DTERLKYVKIRSKKLSKFWGRDMFIGANVVLPAGYNN...QTRYPVIYHQNHWAGADGAYGYGTNKKFTAAWNAGEIPATNKTAARPAPKFIMVSFRHES    297

XP_003297111.1    PFYDDSYAVNSPNLGPYGDALNDELVPHLDMMFNTIAQPYARIQEGGSTGGWAAAASVIFRPDLYGACFASYPDSLDFHRHQDNNLYEQAKAYVRNGLPV    176
XP_003305055.1    PYYDDSYAVNTANLGPYGDAINDELIPYIEGRFKTIRAPYARIQDGGSTGGWESIANVIYRPDLFGACFSSYPDSLDFHRHQDIELYTASNAYQRADGSF    397
XP_001930965.1    PYYDDS..........................FKTIRAPYARIQDGGSTGGWESVANVIYRPDLFGACFSSYPDSLDFHRHQDIELYTASNAYQRADGSF    371

XP_003297111.1    PSNRKVDVDGVERVKVTMEAENHWELTFGTSGRSALQTDVWFAVFSVQGLNGYPLAAWDKITGEIYPDVVELWKPMDLTHHILSNWNSKLNLGEVLRGRI    276
XP_003305055.1    VPSIRTHVNGTEVVESTVAQENHWELSFGTKSRSFNQWDVWNAVFGVQGYNNYPLEPWDKVTGEIYPEAVEHWKPFDLSNYVVANFNSPRDLGTALAGRI    497
XP_001930965.1    VPSIRTHVNGTEVVEATVAQENHWELSFGTSSRSFNQWDVWNAVFGVQGYNNYPLEPWDKVTGEIYQEAVEHWKPFDLSNYVVSNFNSSRNLGTALAGRI    471

XP_003297111.1    HVYVGGVDSYYLDEGVEQFRMRVTEKGGPEWANVTVLPGKAHGGVYNLLPKWTYLDLLLQWVAHHAPGGKTPLSDECTGGKSRGNLWENVLAFGGRQAAL    376
XP_003305055.1    FVYVGTWDNYYLNEGVMEFQKRTDAVGGSGWANVTILPEKPHGGNYQARETWNYLELVEKWVLDHSPTGPAPLSPSSIDPSTRGNIWDDVIQTGGRKAAM    597
XP_001930965.1    FVYVGTWDNYYLNEGVMEFQKRTDAAGGSGWANVTILPEKPHGGNYQARETWDYLELVEKWVLDHSPSGPAPLSPSSIDPSTRGNIWDDVIRNGGRKAAV    571

XP_003297111.1    ARQSPPHIDMYPSHQSDGRLHAEPQASVGRWDPGVALKAQWLVDGKVSKDNVFDVKQRETLTFSGNAPGRTLQLVVTGTKRDYIEETRRSNVITLG      472
XP_003305055.1    KRQAAPKIATKQAKVGEN.....VTASVGRWDPGVKLTAQFVLNNKP.AYEAFCVKQGATVQYTPTAKG.HVQLFVTGQKRNYVTETRKSNRVLVG      686
XP_001930965.1    KRQGAPKIATKQAKVGQN.....VTASVGRWDPGVKLTAQFVLNSKP.ACAAFSVQQCATVQYTPTAKG.HIQLLVTGQKRNYVTETRKSNSVLVG      660
```
